# Novel Thiazolylimidazole Hybrids as Promising Antileishmanial Agents: Rational Design and Biological Evaluation

**DOI:** 10.3390/pathogens15050544

**Published:** 2026-05-18

**Authors:** Cristoper Ramírez-Sandoval, María Elena Campos-Aldrete, María Estela Meléndez-Camargo

**Affiliations:** 1 Laboratorio de Investigación 7 y 10, Departamento de Química Orgánica, Escuela Nacional de Ciencias Biológicas, Instituto Politécnico Nacional, Prolongación de Carpio y Plan de Ayala S/N, Colonia Santo Tomás, Mexico City C.P. 11340, Mexico; christunning1@gmail.com; 2 Laboratorio de Toxicología de Productos Naturales, Departamento de Farmacia, Escuela Nacional de Ciencias Biológicas, Campus Zacatenco, Instituto Politécnico Nacional, Av. Wilfrido Massieu s/n Col. Zacatenco, Mexico City C.P. 07738, Mexico; emelendezc@hotmail.com

**Keywords:** *Leishmania mexicana*, leishmania promastigotes, thiazolyl–imidazole hybrids, thioredoxin reductase, miltefosine resistance

## Abstract

Leishmaniasis remains a major neglected tropical disease with limited therapeutic options, challenged by drug toxicity and emerging resistance to current treatments like miltefosine. In this study, a virtual library of approximately 150 azole-derived compounds was screened in silico to identify promising thiazole and imidazole scaffolds, leading to the rational design of novel hybrid molecules. Molecular docking against thioredoxin reductase (PDB ID: 4CBQ), a key enzyme in the redox metabolism of *Leishmania mexicana*, showed improved binding affinity compared to miltefosine, with compound **3f** showing the most favourable interaction profile. Among the synthesized series **3a**–**f**, compound **3f** (4-NO_2_Ph) exhibited the most favourable predicted binding parameters within the series (∆G = −16.08, Ki = 0.0019 nM). Biological evaluation was performed against *L. mexicana* promastigotes as an early-stage phenotypic screening model to identify active compounds with potential relevance during the initial infective phase, and a markedly improved in vitro inhibitory effect (IC_50_ = 22.41 µM) compared to miltefosine (IC_50_ = 132.42 µM), representing a six-fold increase in molar potency. Furthermore, hybrid thiazolyl–imidazole systems (series **3**) consistently outperformed single-core analogues, likely due to enhanced molecular planarity and lipophilicity provided by the imine linkage. Cytotoxicity assays in Vero cells revealed a high safety margin for the lead compounds, with compound **3f** achieving a Selectivity Index (SI) of around 89, significantly outperforming the reference drug. Acute toxicity studies (LD_50_) in murine models further confirmed the safety profile, with values exceeding 2000 mg/kg for the most active derivatives. These findings identify thiazolyl–imidazole hybrids as promising early-stage scaffolds for antileishmanial drug discovery, particularly for early infection/prophylactic screening.

## 1. Introduction

Leishmaniasis remains a major neglected tropical disease (NTD) affecting millions of people worldwide, according to the most recent World Health Organization assessments (2024–2025) [1,2,3]. It is caused by protozoan parasites of the genus *Leishmania*, with visceral (VL) and cutaneous (CL) leishmaniasis being the primary clinical manifestations [1]. These parasites infect mammalian macrophages, where they survive and replicate within the phagolysosomal compartment. Clinical progression is heavily influenced by parasite-induced immunosuppression and variability in diagnostic and therapeutic efficacy [4,5].

**Figure 1:**
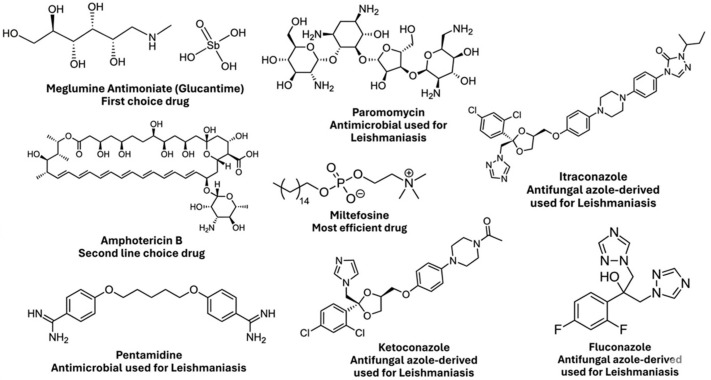
Drugs used for the treatment of Leishmaniasis.

Despite extensive research into immune responses and host–parasite interactions, an approved human vaccine remains unavailable [6]. Current chemotherapy (Figure 1) remains limited, often toxic, and difficult to access in endemic regions. Pentavalent antimonials, such as meglumine antimoniate (Glucantime), were long considered first-line treatments despite their association with cardiotoxicity [7] and pancreatitis [8] and have been linked to increasing reports of clinical resistance [9]. Alternative agents such as amphotericin B exhibit high toxicity, whereas paromomycin shows variable, strain-dependent efficacy [10]. Although pentamidine is effective parenterally, it causes significant metabolic adverse effects and lacks an oral formulation [11]. More recently, miltefosine [12] has gained prominence for its oral administration; however, its clinical utility is compromised by the risk of teratogenicity and the emergence of resistance mechanisms [13,14].

Given these therapeutic challenges, identifying new chemical entities that target essential metabolic pathways has become a priority. In this context, thioredoxin reductase (PDB ID: 4CBQ), selected as a relevant molecular target for its essential role in parasite redox metabolism, was used as a model system to explore potential ligand–enzyme interactions in *Leishmania* species [15].

Building upon the chemical diversity of established antiparasitic scaffolds, a virtual library of 150 azole-derived compounds was screened, identifying thiazole [16] and imidazole [17] nuclei as privileged structures for drug design. In this work, we report the rational design and biological evaluation of a series of novel thiazolyl–imidazole hybrids incorporating specific functional groups to optimize leishmanicidal activity [18,19].

By combining these two pharmacophores, we sought to explore a novel chemical space and enhance potency against the infective promastigote stage of *L. mexicana*, while maintaining a favourable preliminary safety profile. This experimental design was conceived as an early-stage screening approach, since promastigotes correspond to the vector-derived infective forms responsible for initiating mammalian infection. Therefore, this model is useful for identifying compounds that may be relevant in early infection or prophylactic contexts.

## 2. Materials and Methods

Reagents and solvents were purchased from Sigma-Aldrich (Mexico City, Mexico). The reactions were monitored via thin-layer chromatography (TLC) on Merck F253 silica gel aluminum sheets. The infrared spectra were recorded using PerkinElmer Spectrum Version 10.4.4. On the other hand, the NMR spectra (^1^H, ^13^C, HMBC, and COSY) were acquired on Bruker instruments (Mexico City, Mexico) operating at 750–189 MHz, 600–151 MHz, and 400–100 MHz. The melting points were determined using an Electrothermal Melting Point IA9100 (Mexico City, Mexico). The biological assay was performed using an Olympus CKX-41 inverted microscope (Olympus Corporation, Tokyo, Japan) and a Beckman Coulter DTX 800 Multimode plate (Mexico City, Mexico) reader. The *Leishmania mexicana* ATCC 50157 (atcc.org/products/50157) was purchased commercially with certification and proliferated in the Schneider medium and supplemented with 10% FBS (fetal bovine serum, by Products^®^), and 1% antibiotic mixture, in vitro^®^, (the antibiotic mixture consisted of penicillin–streptomycin solution containing 10,000 U/mL penicillin and 10,000 µg/mL streptomycin in 0.85% NaCl, used at 1% *v*/*v* to obtain final concentrations of 100 U/mL penicillin and 100 µg/mL streptomycin) DMSO [20], and deuterated solvents as DMSO-d6 and CDCl_3_ were employed by NMR.

### 2.1. In Silico Affinity Mapping Procedure

For this study, the therapeutic target selected was thioredoxin reductase from *Leishmania* (PDB ID: 4CBQ) [15], due to its essential role in the parasite’s antioxidant defence system and redox metabolism. The crystallographic structure was retrieved from the Protein Data Bank and carefully prepared in AutoDock Tools 1.5.6. Preparation involved removing all crystallographic water molecules, adding polar hydrogens to restore correct protonation states, and assigning Gasteiger partial charges to ensure an accurate electrostatic representation of the macromolecule. The structure was then converted to PDBQT format for docking studies.

Ligand preparation was performed using ChemDraw Proffessional 15.0 3D (Perkin Elmer, Shelton, CT, USA). All ligands corresponding to series **1**, **2**, and **3** were first sketched and optimized to their minimum-energy conformations using molecular mechanics, followed by geometry minimization. The resulting structures were exported in PDB format, subjected to charge assignment and torsional flexibility analysis, Then These were converted to PDBQT files using AutoDock Tools 1.5.6. This workflow ensured that all rotatable bonds were properly defined and that the ligand conformational space was maximally explored.

Docking simulations were performed using AutoDock 4.2.5, and a two-step approach was implemented. First, blind docking was conducted using a 160 Å grid box encompassing the entire protein to identify potential binding regions and evaluate non-specific binding preferences. The Lamarckian genetic algorithm (LGA) was employed with 100 independent runs per ligand, providing statistical robustness and minimizing bias in conformational sampling. Once the binding region was identified, a targeted docking was performed, which restricted the search space to the catalytic site surrounding the flavin adenine dinucleotide (FAD) cofactor. In this case, the grid box was reduced to 60 Å to increase the resolution of the predicted interactions and to capture specific ligand–enzyme contacts.

For each docking experiment, binding free energy (ΔG, kcal/mol), inhibition constant (Ki, nM), and partition coefficient (LogP) were calculated. These values were used to rank the compounds relative to miltefosine, as the reference standard. In addition to numerical scoring, docking poses were examined for consistency and reproducibility across multiple runs to validate the robustness of the predicted binding modes.

Visualization and post-docking analysis were conducted in Discovery Studio 2025 (BIOVIA). Protein–ligand complexes were analyzed to identify hydrogen bonds, π–π stacking, π–cation interactions, and hydrophobic contacts within the active site.

The spatial orientation of each ligand relative to the FAD cofactor and catalytically relevant residues was carefully examined, providing insights into potential mechanisms of inhibition. Structural features such as steric hindrance, electron-withdrawing substituents, and lipophilic contributions were correlated with docking scores to establish structure–activity relationships.

The *in silico* strategy enabled the prediction of binding affinities and provided a molecular rationale for the observed biological activity. The integration of docking outcomes with physicochemical descriptors such as LogP reinforced the importance of lipophilicity, electronic effects, and steric contributions in modulating enzyme–ligand interactions, highlighting the potential of azole derivatives as optimized candidates against leishmaniasis.

It is important to note that the docking analysis provides a predictive approximation of ligand–enzyme interactions and should not be interpreted as definitive evidence of a specific mechanism of inhibition.

The docking protocol was validated by redocking a co-crystallized ligand/cofactor from the crystallographic structure of thioredoxin reductase. The crystallographic ligand was removed from the receptor, prepared using the same ligand-preparation workflow as for the synthesized compounds, and redocked into a grid box centred on its original binding coordinates. The best-ranked redocked pose was superimposed onto the experimental crystallographic orientation after protein alignment, and the heavy-atom root-mean-square deviation was calculated. An RMSD value ≤ 2.0 Å was considered indicative of an acceptable reproduction of the crystallographic binding mode.

### 2.2. General Procedure for Synthesis of 2-Amino-4-arylthiazoles (***1***)

In a flask equipped with a magnetic stirrer and reflux condenser, thiourea (1.0 eq, 13.13 mmol) was dissolved in a minimal volume of absolute methanol. Subsequently, the corresponding α-haloketone (1.0 eq, 13.13 mmol) was added, and the reaction mixture was heated under reflux with constant stirring for 4–24 h. The progress of the reaction was monitored by thin-layer chromatography (TLC) until the starting reagents were completely reduced.

After completion, the reaction mixture was allowed to cool to room temperature, then placed in an ice bath to induce crystallization. The resulting solid was collected by vacuum filtration, washed with cold water and hexane to remove residual impurities (e.g., acetophenone derivatives), and dried under reduced pressure. The crude product was recrystallized from ethanol to afford the corresponding 2-amino-4-arylthiazole derivatives.

The spectroscopic data for compounds **1a**–**1e** were consistent with previously reported data for 2-amino-4-arylthiazoles [16]. As compounds **1a**–**1e** have been previously reported, only compounds **1f** and **2** are included here as representative precursors used for the preparation of series **3**. Their corresponding spectroscopic data are provided in the Supplementary Information.

#### 2.2.1. Data of 2-amino-4-phenyl Thiazole (**1a**)

White solid. Melting point: 150 °C, Yield: 90%. IR (KBr, ν, cm^−1^): 3394–3270 (N-H, NH_2_), 3127 (C-H aromatic), 1633 (C=N, Tz), 1532, 1518 (C=C aromatic), 710 (C-S aromatic) ^1^H NMR (DMSO-d_6_, ppm) δ 7.00 (s, 1H), 7.07 (bs, 2H), 7.25 (m, 1H), 7.36 (m, 2H, 8), 7.79 (d, *J* = 7.5 Hz, 2H). ^13^C NMR (DMSO-d_6_, ppm) δ101.3, 125.4, 126.9, 128.2, 134.8, 149.8, 168.0.

#### 2.2.2. Data of 2-amino-4-p-tolyl Thiazole (**1b**)

White solid. Melting point: 135 °C. Yield: 83%. IR (KBr, ν, cm^−1^): 3298 (N–H, NH_2_), 3113 (C–H aromatic), 2920–2850 (C-H, CH_3_ group), 2225 (C≡N), 1639 (C=N Tz), 1500–1450 (C=C aromatic), 750–700 (C–H aromatic), 670 (C–S Tz). ^1^H and ^13^C NMR signals [16].

#### 2.2.3. Data of 2-amino-4-fluorophenyl Thiazole (**1c**)

Light beige solid. Melting Point: 109 °C. Yield: 75%. IR (KBr, ν, cm^−1^): 3327, 3278 (N-H, NH_2_), 3116 (C-H aromatic), 1633 (C=N Tz), 1589–1511 (C=C aromatic), 1118–1033 (C-F), 1436–1414 (C-H aromatic), 1189 (C-F aromatic), 850–700 (C-H aromatic), 665 (C-S Tz). ^1^H NMR (DMSO-d6 ppm) δ 7.63–7.56 (m, 2H), 7.15–7.07 (m, 2H, d, *J =* 7.9 Hz), 7.00 (s, 1H), 6.13 (s, 2H). ^13^C NMR (DMSO-d6, ppm) δ 166.91, 164.17, 162.20, 131.93, 131.14, 130.76, 128.91, 128.83, 115.92, 115.75.

#### 2.2.4. Data of 2-amino-4-chlorophenyl Thiazole (**1d**)

White solid. Melting point: 168 °C. Yield: 85% IR (KBr, ν, cm^−1^): 3421, 3268 (N-H, NH_2_), 3120 (C-H aromatic). 1633 (C=N Tz), 1500-1450 (C=C aromatic), 1436-1414 (C-H aromatic), 800-600 (C-Cl). ^1^H and ^13^C NMR signals [16].

#### 2.2.5. Data of 2-amino-4-cyanophenyl Thiazole (**1e**)

White solid. Melting point: 177 °C. Yield: 87%. IR (KBr, ν, cm^−1^): 3405-3243 (N-H, NH_2_), 3093 (C-H aromatic), 2217 (Nitrile group Aromatic), 1627 (C=N, Tz), 1500–1450 (C=C aromatic), 800–700 (C-H aromatic). ^1^H and ^13^C NMR signals [16].

#### 2.2.6. Data of 2-amino-4-nitrophenyl Thiazole (**1f**)

Yellow solid. Melting point: 159 °C. Yield 72%. IR (ATR, ν, cm^−1^): 2988 (N–H, NH_2_), 1640 (C–N aromatic), 1536, 1499, 1408, 1106, 1039 (Tz), 841, 720, 661 (C–S, Tz) cm^−1^. ^1^H NMR (DMSO-d6, ppm) δ 8.26–8.20 (m, 2H, Ar-H), 8.08–8.03 (d, *J* = 7.8 Hz, 2H, Ar-H), 7.24 (s, 1H, H-5 thiazole), 5.90 (bs, 2H, NH_2_). ^13^C NMR (DMSO-d6, ppm) δ 168.31, 147.77, 142.44, 133.86, 128.29, 127.22, 124.09.

#### 2.2.7. Data of 3-(N)-phenacyl-2-methyl-5-nitroimidazole (**2**)

White solid. Melting Point: 195–196 °C. Yield: 90%. IR (ATR, ν, cm^−1^): 3142, 3071 (C–H aromatic), 2988, 2938 (C–H aliphatic), 1688 (C=O), 1597 (C=N/C=C aromatic), 1538, 1500, 1327 (NO_2_), 1450, 1392 (C–N/C–H bending), 1290, 1228, 1137 (C–N), 985, 837, 753, 684 (C–H aromatic, out-of-plane), 633, 584. ^1^H NMR (DMSO-d_6_, ppm) δ 8.28 (s, 1H, H-4 imidazole), 8.10–8.06 (m, 2H, Ar–H), 7.79–7.73 (m, 1H, Ar–H), 7.64 (t, *J* = 7.6 Hz, 2H, Ar–H), 5.94 (s, 2H, N–CH_2_), 2.28 (s, 3H, CH_3_). ^13^C NMR (DMSO-d_6_, ppm) δ 192.76, 147.26, 146.68, 145.75, 145.18, 134.80, 134.49, 129.45, 128.69, 123.78, 119.45, 53.84, 14.22, 12.94.

### 2.3. General Procedure for Synthesis of the 1-(2-Methyl-5-nitro-1H-imidazol-1-yl)-2-phenyl-N-(4-phenylthiazol-2-yl)ethan-1-imine (Series ***3***)

The corresponding 2-amino-4-arylthiazole derivatives (**1a**–**f**, 1.0 eq.) were dissolved in absolute methanol, and triethylamine (5.0 eq.) was added dropwise under continuous stirring to generate a basic medium. Subsequently, 3-(N-phenacyl)-2-methyl-5-nitroimidazole (2, 1.0 eq.) was added, and the reaction mixture was heated at reflux for 4–24 h. The progress of the reaction was monitored by thin-layer chromatography (TLC).

The reaction mixture was allowed to cool to room temperature, and the precipitated solid was collected by vacuum filtration, washed with cold methanol, and dried under reduced pressure. The crude product was recrystallized from dichloromethane (or a suitable solvent) to afford the corresponding imine derivatives (**3a**–**f**) as pale yellow to off-white solids.

The structures of the synthesized compounds were confirmed by infrared spectroscopy (IR), ^1^H and ^13^C nuclear magnetic resonance (NMR), and two-dimensional NMR experiments (HMBC). Chemical shifts (δ) are reported in ppm relative to residual solvent signals (DMSO-d_6_). Multiplicities are described as s (singlet), d (doublet), t (triplet), and m (multiplet), and coupling constants (J) are reported in Hz. Signal assignments were based on ^1^H and ^13^C NMR data and supported by HMBC and COSY correlations.

#### 2.3.1. Data of 1-(2-methyl-5-nitro-1H-imidazol-1-yl)-2-phenyl-N-(4-phenylthiazol-2-yl)ethan-1-imine (**3a**)

Pale beige solid. Melting point: 258 °C. Yield: 49%. IR (ATR, ν, cm^−1^): 3142 (C–H aromatic/heteroaromatic), 2987 (C–H aliphatic), 1687 (C=N imine), 1598 (C=C aromatic), 1538 (N–O, NO_2_), 1501 (C=C aromatic), 1450 (C–H aromatic), 1393 (N–O, NO_2_), 1137 (C–N), 986 (C–H), 837, 754 (C–H aromatic), 634 (Tz). ^1^H NMR (DMSO-d6, ppm) δ 8.22 (d, *J* = 1.4 Hz, 1H), 8.08–8.04 (m, 2H), 7.80–7.40 (m, 8H, Ar–H), 7.27 (s, 1H, imine-CH), 5.86 (s, 2H), 2.27 (d, *J* = 1.7 Hz, 3H). ^13^C NMR (DMSO-d6, ppm) δ 194.99, 146.13, 144.59, 139.01, 135.30, 135.24, 134.13, 133.51, 130.58, 130.04, 129.59, 128.96, 128.52, 127.90, 124.20, 51.28, 13.64.

#### 2.3.2. Data of 1-(2-methyl-5-nitro-1H-imidazol-1-yl)-2-phenyl-N-(4-(*p*-tolyl)thiazol-2-yl)ethan-1-imine (**3b**)

Reddish solid. Melting point: 261 °C. Yield: 45%. IR (ATR, ν, cm^−1^): 3130 (C–H aromatic/heteroaromatic), 1683 (C=N imine), 1607 (C=C aromatic), 1538 (N–O, NO_2_), 1499 (C=C aromatic), 1394 (N–O, NO_2_), 1186 (C–N), 996 (C–H), 832 (C–H aromatic), 669 (Tz). ^1^H NMR (DMSO-d6, ppm) δ 8.73 (s, 1H), 8.23 (s, 1H), 7.95–7.30 (m, 8H, Ar–H), 5.82 (s, 2H, CH_2_), 2.42 (s, 3H, Ar–CH_3_), 2.25 (s, 3H, imidazole-CH_3_). ^13^C NMR (DMSO-d6, ppm) δ 194.10, 192.79, 149.99, 148.22, 148.01, 147.44, 146.13, 146.59, 145.01, 139.47, 134.97, 134.01, 133.51, 132.72, 130.58, 130.04, 129.61, 128.96, 127.97, 124.20, 51.28, 21.26, 13.64.

#### 2.3.3. Data of 1-(2-methyl-5-nitro-1H-imidazol-1-yl)-2-phenyl-N-(4-(*p*-fluoro)thiazol-2-yl)ethan-1-imine (**3c**)

Pale white solid. Melting point: 271 °C. Yield: 51%. IR (ATR, ν, cm^−1^): 3129 (C–H aromatic/heteroaromatic), 1683 (C=N imine), 1606 (C=C aromatic), 1537 (N–O, NO_2_), 1499 (C=C aromatic), 1393 (N–O, NO_2_), 1185 (C–N), 995 (C–H), 832 (C–H aromatic), 677 (Tz). ^1^H NMR (DMSO-d6, ppm) δ 8.23 (s, 1H), 7.95–7.20 (m, 9H, Ar–H), 5.82 (s, 2H, CH_2_), 2.25 (s, 3H, CH_3_). ^13^C NMR (DMSO-d6, ppm) δ 192.16, 146.61, 145.76, 145.45, 132.02, 130.60, 130.20, 129.98, 129.60, 129.20, 128.92, 128.77, 127.68, 123.79, 53.57, 12.89.

#### 2.3.4. Data of 1-(2-methyl-5-nitro-1H-imidazol-1-yl)-2-phenyl-N-(4-(*p*-chloro)thiazol-2-yl)ethan-1-imine (**3d**)

White solid. Melting point: 263 °C. Yield: 60%. IR (ATR, ν, cm^−1^): 2982 (C–H aliphatic), 1684 (C=N imine), 1606 (C=C aromatic), 1537 (N–O, NO_2_), 1499 (C=C aromatic), 1393 (N–O, NO_2_), 1185 (C–N), 995 (C–H), 832 (C–H aromatic), 677 (Tz). ^1^H NMR (DMSO-d6, ppm) δ 8.23 (s, 1H), 7.95–7.30 (m, 9H, Ar–H), 6.99 (s, 1H), 5.83 (s, 2H, CH_2_), 2.42 (s, 3H, CH_3_). ^13^C NMR (DMSO-d6, ppm) δ 192.16, 146.61, 145.76, 145.45, 132.02, 130.60, 130.20, 129.98, 129.60, 129.20, 128.92, 128.77, 127.68, 123.79, 53.57, 12.89.

#### 2.3.5. Data of 1-(2-methyl-5-nitro-1H-imidazol-1-yl)-2-phenyl-N-(4-(*p*-nitro)thiazol-2-yl)ethan-1-imine (**3f**)

Light yellow solid. Melting point: 254 °C. Yield: 54%. IR (ATR, ν, cm^−1^): 3129 (C–H aromatic/heteroaromatic), 1683 (C=N imine), 1537 (N–O, NO_2_), 1499 (C=C aromatic), 1393 (N–O, NO_2_), 1185 (C–N), 994 (C–H), 832 (C–H aromatic), 677 (Tz). ^1^H NMR (DMSO-d6, ppm) δ 8.24 (s, 1H), 8.10–7.60 (m, 8H, Ar–H), 5.88 (s, 2H, CH_2_), 2.28 (s, 3H, CH_3_). ^13^C NMR (DMSO-d6, ppm) δ 192.69, 170.20, 147.70, 146.61, 145.85, 143.20, 134.76, 134.57, 129.44, 129.11, 128.66, 128.55, 127.44, 126.76, 124.42, 123.69, 53.69, 12.88.

### 2.4. Biological Evaluation

#### 2.4.1. In Vitro Biological Assays

##### Determination of Leishmanicidal Activity

The antiproliferative activity of the studied compounds was evaluated *in vitro* against promastigote cultures of *Leishmania mexicana* ATCC 50156 using a simultaneous multipanel treatment design based on metabolic activity. The assay is described as a promastigote-based phenotypic screening model. This assay was selected as an initial phenotypic screening model to evaluate the parasite’s response at an early infective stage.

The series **3** hybrids were tested, with miltefosine (MTF) as the reference drug, and the precursors (series **1** and compound **2**) were also included for comparison.

Working solutions were prepared (10 mg/mL in DMSO) with ≤1% DMSO [20] and frozen until use. From these stock solutions, serial dilutions were prepared in culture medium to generate concentration–response curves for IC_50_, as well as to determine and record MIC as a complementary qualitative viability endpoint based on the visual resazurin response. Several dilutions were prepared, yielding seven concentrations [200-3 µg/mL] in Schneider medium, which were supplemented with 10% FBS (fetal bovine serum) and 1% antibiotic mixture, in vitro^®^. *Leishmania mexicana* promastigotes (ATCC 50156) were maintained and expanded in supplemented culture medium containing 10% FBS and 1% penicillin–streptomycin solution at 25–30 °C. Before compound exposure, parasite density was determined, and the inoculum was adjusted to 1 × 10^6^ parasites/well for the antiproliferative assay [21]. Promastigote populations (1 × 10^6^ cells) were treated with test compounds for 24 h, after which parasite growth was assessed microscopically. Subsequently, resazurin (10 μL/well) was added as a metabolic indicator, and the plates were incubated at 37 ± 2 °C until a colour shift from blue to fluorescent pink was observed.

In the present study, this parameter was used as a complementary response activity endpoint, derived from the resazurin/Alamar Blue assay. MIC was defined as the lowest tested concentration at which the blue-to-pink colorimetric transition was visually inhibited, indicating a marked reduction in parasite metabolic activity. IC_50_ values were considered the principal quantitative parameter for comparing antileishmanial activity.

MIC values were recorded as the lowest tested concentration, initially expressed in µg/mL, at which the blue-to-pink colorimetric transition of resazurin was visually inhibited. These values were subsequently converted to µM using the molecular weight of each compound, according to the equation: MIC (µM) = [MIC (µg/mL) × 1000]/molecular weight (g/mol). Absorbance was then measured, and the data were analyzed in Microsoft Excel 365 to generate graphs and perform statistical analysis. The inhibitory concentration was finally determined using the PROBIT method [22]. All biological assays were performed as three independent experiments, each carried out in triplicate (n = 9). Results are expressed as the mean ± standard deviation (SD). The IC_90_ values were estimated based on the experimental IC_50_ results assuming a standard Hill slope (H = 1.0).

##### Cytotoxicity Assays in Vero Cells

Cytotoxicity was evaluated using an *in vitro* model based on Vero cells (African green monkey kidney cells, ATCC CCL-81). Cells were maintained in DMEM supplemented with 2% fetal bovine serum under a humidified atmosphere of 5% CO_2_ at 37 °C until an appropriate cell density was reached for the assay. Prior to experimentation, cell monolayers were washed with phosphate-buffered saline (PBS) to remove residual serum and then detached with trypsin. The resulting cell suspension was gently centrifuged, resuspended in fresh medium, and cell counting was performed using a Neubauer hemocytometer according to the trypan blue exclusion method, ensuring the viability of the cell population employed.

For the assay, cells were seeded into 96-well plates at a density of 3 × 10^5^ cells per well and allowed to adhere for 24 h prior to treatment. After cell adhesion, serial dilutions of the tested compounds were prepared starting from an initial concentration of 800 µg/mL, using culture medium as diluent, and added to the wells to evaluate cellular responses over a broad concentration range. Cells were then exposed to the compounds for 24 h. Each experimental condition was assessed in triplicate, including appropriate controls to normalize metabolic response and to exclude potential interference of the chemical system with the detection method.

At the end of the 24 h treatment period, cell viability was determined using a resazurin-based colorimetric assay, a redox-sensitive indicator of cellular metabolic activity. Resazurin solution was added to each well, and the plates were incubated until stable conversion of the dye was observed. The metabolic reduction of resazurin to resorufin, mediated by intracellular diaphorase-type enzymes, generated a quantifiable signal proportional to the number of viable cells. Absorbance readings were obtained using a microplate reader, with measurement conditions selected to avoid signal saturation or optical interference.

The absorbance values were normalized relative to growth control and expressed as percentage of cell viability. Dose–response curves were constructed from these data and analyzed using a PROBIT statistical model, allowing the estimation of CC_50_ values. The selectivity index was calculated according to the following equation: SI = CC_50_/IC_50_.

#### 2.4.2. *In Vivo* Evaluation of Acute Toxicity

##### Animals Care and Housing

Adult female NIH mice of 30 ± 5 g of body weight (b.w.) for the acute toxicity study were used. Seven days before experimentation, the animals were acclimatized in a vivarium at laboratory conditions (temperature 25 ± 2 °C, light/dark cycles of 12 h, relative humidity 50 ± 5%). Rodent’s standard diet and water were available ad libitum.

The use and care of experimental animals was carried out in accordance with national and international guidelines on the welfare of experimental animals [23,24] and with institutional ethical committee approval (CEI-ENCB-004/2015).

##### Determination of Medium Letal Dose (LD_50_) 

The procedure for determining the lethal dose 50 (LD_50_) of compounds **3a**–**f** was performed in accordance with OECD guideline 423 [25]. Compounds were administered as a single oral dose after an 8 h fast, with water available ad libitum. A total of 84 adult female NIH mice were used for the acute oral toxicity study. Five compounds were evaluated at four dose levels: 250, 500, 1000, and 2000 mg/kg b.w., with four animals per dose group (4 × 5 × 4). A shared vehicle-control group of four animals received vehicle-only treatment under the same experimental conditions, resulting in a total of 84 animals. This design avoided using separate vehicle-control groups for each compound and reduced the total number of animals required. Administration was performed per os, in all groups. All animals were observed during the next three hours post-administration and daily during 14 days to register any change in skin and fur; eyes and mucous membranes; renal, respiratory, circulatory, autonomic, and central nervous systems; and somatic motor activity and behaviour patterns, as well as observations of diarrhea, lethargy, sleep, coma, and death, according to the Organization for Economic Cooperation and Development (OECD) guideline 423 [25].

For compounds **3a**, **3b**, and **3f**, the main signs observed during the first hours included piloerection, fasciculation, tremors, lethargy, and hypothermia. For compounds **3c** and **3d**, prostration, seizures, severe fasciculations, eyelid ptosis, and marked hypothermia were observed. No additional significant behavioural changes or signs of delayed toxicity were observed during the 14-day post-treatment observation period.

In relation to the therapeutic benefit-toxicity balance, the inherent toxicity of nitro-containing compounds (-NO_2_) has been reported, leading to the inclusion of *in vitro* toxicological characterization. Compound **2** was assessed for genotoxicity and showed no genotoxic activity in human lymphocytes using the comet assay [26].

### 2.5. Statistical Analysis

Data were analyzed using a one-way analysis of variance (ANOVA) followed by Dunnett’s post hoc test to evaluate significant differences between the synthesized treatments and the reference drug (Miltefosine). Model robustness was verified through residual analysis, including assessments of normality (Gaussian distribution) and homoscedasticity. All statistical tests were performed at a 95% confidence level (*p* < 0.05).

## 3. Results

### 3.1. In Silico Analysis

For the structure–activity relationship analysis, a virtual library of approximately 150 azaheterocyclic compounds was assembled, comprising scaffolds previously associated with antimicrobial, anti-inflammatory, antineoplastic, and antiparasitic activities.

**Figure 2:**
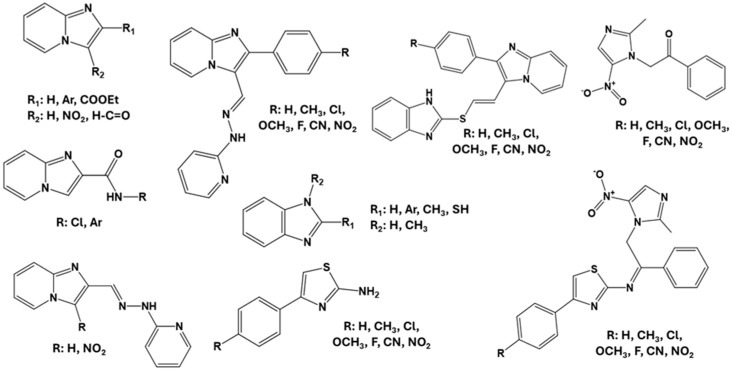
Possible antiparasitic azaheterocycles as a metabolic disruptor, in consideration of the pharmacological target, thioredoxin reductase (4CBQ).

Diverse substituents were incorporated to explore the influence of lipophilic and electronic properties on membrane permeation and biological activity. Among the therapeutic targets evaluated for potential antiparasitic mechanisms, oxidoreductases involved in redox metabolism were selected, including thioredoxin reductase (PDB: 4CBQ) [15] and trypanothione reductase (PDB: 1BZL) [27,28,29]. Molecular docking studies were performed to assess the interaction profiles of the designed compounds related to reference heterocyclic scaffolds, including benzimidazole, thiazole, imidazole, and imidazo[1,2-*a*]pyridine derivatives [30] (Figure 2). The 4CBQ protein was selected due to the presence of thiol-like domains relevant to the redox metabolism of parasites of the genus *Leishmania* [31]. While thiol-like domains are present in various biological systems, the study focused on evaluating interactions between the designed compounds and this specific enzyme using molecular docking as an initial screening approach.

**Figure 3:**
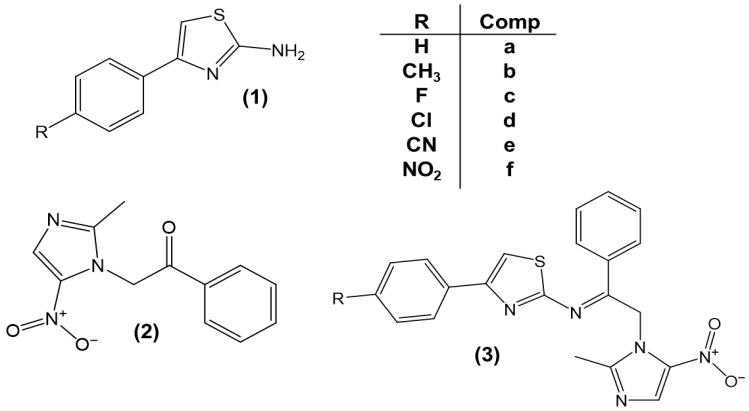
Chemical structures of the selected azolic compounds (**1a**–**f**, **2**, and **3a**–**f**) evaluated as potential antiparasitic agents.

The compounds shown in Figure 3 incorporate key chemical building blocks that favour enhanced recognition within the catalytic site of the 4CBQ protein, suggesting potential leishmanicidal activity. Docking results were interpreted comparatively based on binding energies and interaction profiles relative to the reference compound, rather than as absolute quantitative measures, supporting the hypothesis of a different binding mode and potentially an alternative mechanism of action [32,33]. Based on these analyses, series 1 (**1a**–**f**), compound **2**, and series **3a**–**f** were selected for further study.

**Figure 4:**
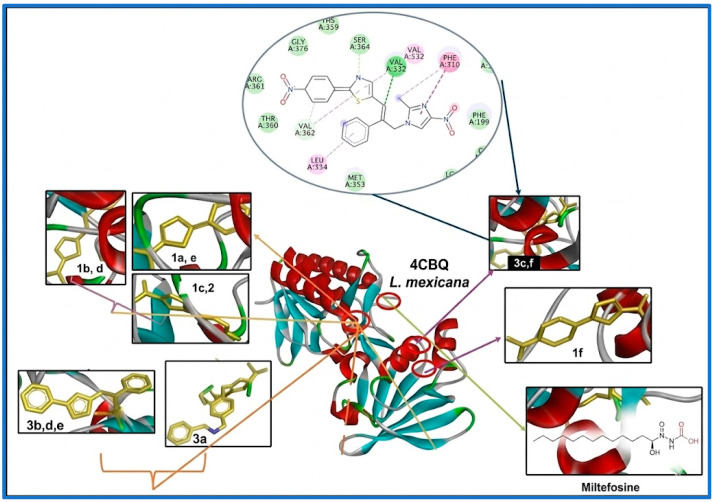
Recognition and affinity by derivatives (**a** = -Ph, **b** = Ph-CH_3_, **c** = Ph-F, **d** = Ph-Cl, **e** = -Ph-CN and **f** = Ph-NO_2_) of the series **1**, compound **2**, series **3** and Miltefosine (reference drug) under study towards 4CBQ protein (thioredoxin reductase).

The predicted binding modes and essential molecular interactions of the selected candidates within the catalytic site are illustrated in Figure 4. This detailed characterization highlights the specific residue contacts and ligand orientations that distinguish the novel hybrid series from the reference drug, miltefosine.

Table 1 summarizes the binding affinity and inhibition parameters obtained from blind molecular docking. Notably, compounds from series **3** exhibited significantly higher binding affinity and lower inhibition constants than the reference drug (miltefosine) and the other evaluated molecules.

The contribution of substituents at the para position (C-4) of the aryl ring (PhNO_2_, PhCN, PhCH_3_, PhCl, Ph, and PhF) was clearly observed. Electron-withdrawing groups such as NO_2_ and CN were associated with the highest binding affinities and lowest inhibition constants, particularly for compounds **3f** and **3e** (NO_2_ and CN), which displayed Ki values in the sub-nanomolar range. In contrast, compounds bearing less electron-withdrawing or neutral substituents (e.g., CH_3_, H, and F) showed reduced binding affinity.

Additionally, these derivatives exhibited greater lipophilicity than the reference drug, potentially enhancing interactions within the hydrophobic regions of the enzyme’s active site. Overall, these results suggest that both electronic effects and lipophilic contributions play a key role in enhancing ligand–protein interactions.

Based on evidence of target affinity for the same receptor region, the miltefosine binding-site coordinates were used to perform *in silico* analysis of targeted molecular coupling across the three series of compounds to assess their competition for the cluster. The results are presented in Table 2.

The results from directed docking further support the influence of substituents on binding affinity. Electron-withdrawing groups such as nitro (NO_2_) and nitrile (CN) showed a stronger contribution to ligand–protein interactions compared to electron-donating groups such as methyl (CH_3_). Accordingly, compounds **3f** and **3e** exhibited the highest binding affinities and the lowest inhibition constants within the series.

In contrast, halogenated derivatives (**3c** and **3d**, bearing F and Cl, respectively) and the unsubstituted compound **3a** displayed reduced affinity toward the catalytic site. Despite these differences, all compounds in series **3** demonstrated significantly improved binding affinity and markedly lower inhibition constants compared with the reference drug, miltefosine. Overall, these findings highlight the critical role of electronic effects in modulating binding interactions within the 4CBQ active site. Based on this evidence, selected compounds such as **3a**–**f** were prioritized for synthesis and subsequent biological evaluation.

Finally, compound **2** (3-N-phenacyl-2-methyl-5-nitroimidazole), despite incorporating a nitro parasitophore group (NO_2_), a lipophilicity-modulating methyl group (CH_3_), and an N-phenacyl moiety capable of steric and charge-transfer interactions, exhibited lower biological activity than compounds from series **1** and **3**. This behaviour suggests that the absence of structural hybridization between thiazole and imidazole scaffolds limits its ability to achieve optimal interactions within the catalytic site.

In contrast, the evaluated azole derivatives, particularly compound **3f**, displayed significantly higher antiparasitic activity than miltefosine, with at least a fivefold increase in potency and markedly lower *in silico* inhibition constants. These results reinforce the importance of structural hybridization and substituent-driven electronic effects in enhancing ligand–protein interactions, supporting thiazolyl–imidazole derivatives as promising candidates for the development of new therapeutic alternatives against leishmaniasis.

Redocking validation showed that the docking protocol reproduced the crystallographic binding orientation of the reference ligand/cofactor, yielding a heavy-atom RMSD of 1.5 Å. This result supports the suitability of the grid definition, ligand-preparation workflow, and docking parameters for subsequent analysis of the synthesized thiazolyl–imidazole derivatives.

### 3.2. Synthesis of Antileishmanial Compounds

#### 3.2.1. Synthesis of 2-Amino-4-phenyl thiazoles [34]

The series of compounds **1a**–**f** was obtained via condensation of the corresponding α-haloketones with thiourea under reflux conditions in methanol, following a Hantzsch-type thiazole synthesis. The reaction proceeded efficiently in a protic medium, affording the desired 2-amino-4-arylthiazole derivatives in good to excellent yields, as summarized in Table 3.

The structural identity of the synthesized 2-amino-4-arylthiazole derivatives was confirmed by their characteristic infrared absorption bands, consistent with those reported for this scaffold. In particular, the presence of the amino group was evidenced by broad absorption bands in the 3300–3200 cm^−1^ region, while the thiazole ring exhibited characteristic C=N stretching vibrations in the range of 1620–1580 cm^−1^, along with C–S stretching bands observed between 750 and 700 cm^−1^.

These spectral features are consistent with previously reported data for para-substituted arylthiazoles, including *p*-chloro, *p*-fluoro, and *p*-tolyl derivatives [34].

Moreover, the literature indicates that these IR patterns are largely independent of the nature of the aromatic substituent and are considered diagnostic of this heterocyclic core [16].

#### 3.2.2. Synthesis of 1-(2-Methyl-5-nitro-1H-imidazol-1-yl)-2-phenyl-N-(4-R-phenylthiazol-2-yl) ethan-1-imine (Series **3**)

A nucleophilic addition reaction from the compounds 2-amino-4-phenyl thiazoles (**1a**–**f**) was carried out with the amino group of series **1** on the carbonyl core of 3-N-phenacil-2-methyl-5-nitroimidazole (**2**), to generate the imine bridge that allowed obtaining the hybrid thiazolylimidazole compounds under basic ethanolic conditions. The synthesis results obtained are presented in Table 4.

Overall, the yields for the analogues of series **3** ranged from 45% to 60%. The exception was compound **3e** (R = CN), which was obtained as a complex mixture that could not be resolved by chromatographic methods. This behaviour is likely associated with the strong electron-withdrawing nature of the nitrile substituent, which may reduce the nucleophilicity of the amine intermediate and destabilize the imine linkage formed during the condensation reaction.

Additionally, the presence of the nitrile group may increase the imine bond’s susceptibility to hydrolysis or promote competing side reactions under the reaction conditions, leading to multiple products. Due to this instability, compound **3e** could not be isolated in pure form or quantitatively obtained and was therefore excluded from further pharmacological evaluation.

### 3.3. In Vitro Studies

The *in vitro* assays were performed to evaluate the antiparasitic and cytotoxic activities of the synthesized compounds using a viability-based detection method. Experiments were conducted in 96-well microplates, testing ten compounds at seven concentrations, along with appropriate controls, including untreated cells and reference drugs. All assays were carried out with inter- and intra-assay replicates to ensure reproducibility. Cell viability was determined after 24 h of treatment using resazurin as a metabolic indicator, and the resulting spectrophotometric measurements were recorded and statistically analyzed. Dose–response curves were generated from the viability data, and IC_50_ and CC_50_ values were calculated using the probit method [22]. Complementarily, IC_90_ values were estimated to assess the concentration required for near-total parasite elimination. As shown in Table 5, the IC_90_ values followed a structure–activity relationship (SAR) trend consistent with the IC_50_ results, reinforcing compound **3f** as the most effective derivative of the series.

#### Antileishmanial Response and Cytotoxicity

The antileishmanial response was determined on *Leishmania mexicana* promastigotes (1 × 10^6^/well) and was consistent with the behaviour observed in the *in silico* study.

Since this assay was designed as an early-stage screening model, the resulting IC_50_ values should be interpreted as preliminary indicators of leishmanicidal potential rather than definitive evidence of efficacy against established intracellular infection. Compounds showing improved potency and selectivity in this model were therefore considered candidates for subsequent assays.

All compound analogues (**1** and **3**) tested were less cytotoxic than the reference drug (MTF), as indicated by the CC50 values in Vero cells, a mammalian cell model, in Table 5.

According to the results presented in Table 5, the influence of *p*-substituted aryl groups (Ph–H, Ph–CH_3_, Ph–F, Ph–Cl, and Ph–NO_2_) is clearly reflected in both antiparasitic activity and selectivity. A consistent structure–activity relationship was observed, in which electron-withdrawing substituents, particularly the nitro group, significantly enhance biological performance. This trend is in strong agreement with the *in silico* results, in which compounds bearing NO_2_ and CH_3_ groups exhibited the highest binding affinities and the lowest inhibition constants toward thioredoxin reductase (4CBQ).

Notably, compound **3f** exhibited the best overall profile, with the lowest IC_50_ value (22.41 µM) and the highest selectivity index (SI ≈ 89), clearly outperforming the reference drug miltefosine (IC_50_ = 132.42 µM; SI ≈ 27). In addition, compound **3f** showed the lowest MIC value as a complementary qualitative viability endpoint based on the visual resazurin response. This represents an approximate six-fold increase in potency, together with a substantially improved safety margin.

Compounds **3b** and **3d** also exhibited favourable activity and selectivity (SI ≈ 21–22), indicating that moderate lipophilic and electronic contributions can still promote effective interactions with the target enzyme. In contrast, unsubstituted or weakly electron-withdrawing derivatives (**3a** and **3c**) showed reduced activity and selectivity, highlighting the critical role of substituent-driven electronic effects in modulating biological response.

Importantly, hybrid thiazolyl–imidazole systems (series **3**) consistently outperformed their single-core counterparts (series **1**).

This behaviour can be rationalized by the presence of the imine linkage, which not only increases molecular planarity and conjugation, improving π–π stacking and electronic delocalization, but also enhances lipophilicity and facilitates additional stabilizing interactions within the enzyme active site. This dual contribution likely explains the improved correlation between docking affinity and *in vitro* activity observed for series **3**.

Overall, these findings support a clear structure–activity relationship in which both electronic effects and scaffold hybridization synergistically enhance antiparasitic potency and selectivity, positioning compound **3f** as a promising lead for further optimization.

### 3.4. In Vivo Studies (Acute Toxicity Study)

The acute oral toxicity assay was incorporated as a preliminary safety-oriented filter within the compound prioritization process. Since the present study was designed as an early-stage antileishmanial screening approach, this assay was used to determine whether the most active hybrids displayed an acceptable acute safety profile before further biological validation.

To determine the lethal dose 50 (LD_50_, dose capable of causing death in 50% of the animals) of the compounds **3a**–**f**, female mice were treated with a single administration and were put into experimental groups for all compounds at different doses of each compound at 250, 500, 1000 and 2000 mg/kg body weight (b.w.) [25], respectively, within the control group (vehicle, water 1 mL/kg b.w.). Acute toxicity is shown in Table 6.

This study demonstrated that the LD_50_ values for compounds **3a** (-H), **3b** (-CH_3_), and **3f** (-NO_2_) are significantly greater than 2000 mg/kg in murine animals, indicating a relatively low risk of toxicity [25]. However, subacute and chronic toxicity studies are still necessary. The **3c** (-F) and **3d** (-Cl) compounds showed an LD_50_ less than 1000 mg/kg b.w., and a CC_50_ (Table 6). The main signs observed in some animals for compounds **3a**, **3b**, and **3f** during the first three hours were piloerection, fasciculation, tremors, lethargy, and hypothermia. For **3c** and **3d**, prostration, seizures, severe fasciculations, eyelid ptosis, and marked hypothermia were observed. The brief mention of signs of toxicity was described.

The pharmacological response of compounds **3f** and **3b** is associated with electronic effects: the conjugative effect through the nitro group (**3f**) towards the core heterocyclic ring favoured metabolic inhibition, and the positive inductive effect of the methyl group over the imine group increased the affinity constant (**3b**). In addition, the lipophilic contribution on **3b** and **3c** suggests good permeation to the target.

The electronic effect of the substituent at the para position of the arylthiazole modulates the interactions between the ligand and the protein: hydrogen-bridge-type interactions mediated by charge transfer, leading to improved activity via enhanced electrostatic interactions. All of these are more active than miltefosine, and compounds **3a** and **3b** are more toxicologically safe.

## 4. Discussion

The development of novel therapeutic alternatives against *Leishmania* species is a global health priority, particularly due to the increasing reports of clinical resistance and significant toxicity associated with the current gold standard, miltefosine. In this study, we successfully integrated computational design and structural hybridization to develop a series of thiazolyl–imidazole derivatives that target the redox defence system of the parasite, specifically focusing on the infective promastigote stage.

The selection of promastigotes aligned with the exploratory scope of the study and an early-infection/prophylactic-screening rationale. Promastigotes are the infective forms transmitted by the vector and initiate infection in the mammalian host before differentiating into intracellular amastigotes. Therefore, evaluating this stage allows the identification of compounds with potential activity during the initial parasite-exposure phase. Thioredoxin reductase is proposed here as a putative molecular target based on docking analysis, rather than as an experimentally validated mechanism of action. Although the redocking validation supports the reliability of the computational protocol, future biochemical assays will be required to confirm direct enzyme inhibition.

The SAR observed in this series appears to depend on a balance between electronic activation, lipophilicity, and scaffold planarity. Compound **3f**, bearing a *p*-NO_2_ group, showed the best profile, likely because the strong electron-withdrawing character of this substituent increases polarization across the arylthiazole–imine system and may favour dipolar, hydrogen-bond acceptor, or electrostatic interactions within the predicted binding region. In contrast, the methyl derivative **3b** may benefit mainly from hydrophobic contributions, whereas the chloro derivative **3d** showed an intermediate profile, probably associated with a balance between lipophilicity and steric effects. The similar behaviour of **3a** and **3c,** reinforces the isosteric character between the -H and -F. Together, these findings indicate that the enhanced activity of series **3** is not only related to the presence of the thiazolyl–imidazole hybrid scaffold, but also to the ability of specific *p*-substituents to modulate electronic distribution, hydrophobic complementarity, and ligand–protein stabilization.

The statistically significant effect of compounds **3f**, **3b**, and **3d** on miltefosine (*p* < 0.05) allows us to conclude that the antileishmanial response is attributable to the contribution of heterocyclic systems rather than to random experimental variation.

A key highlight of this research is the superior performance of the hybrid systems (series **3**) over their single-core counterparts (series **1** and **2**). This synergistic effect can be attributed to the incorporation of the imine linkage, which increases molecular planarity and conjugation. As suggested by our *in silico* models, this structural rigidification facilitates additional stabilizing interactions—such as π-π stacking and hydrogen bonding within the hydrophobic pockets of the 4CBQ enzyme, which is essential for the parasite’s antioxidant defence.

Beyond potency, the safety profile is a determining factor for any new leishmanicidal candidate. Compound **3f** exhibited a remarkable Selectivity Index (SI) of approximately 89, which is three times higher than that of miltefosine (SI ≈ 27). This high selectivity indicates that our hybrids can effectively distinguish between the parasite’s redox machinery and mammalian host cell functions. Furthermore, the *in vivo* acute toxicity studies corroborated these findings, as compound **3f** demonstrated an LD_50_ greater than 2000 mg/kg, a value far exceeding the active therapeutic concentrations. The absence of delayed toxicity during the 14-day observation period supports the prioritization of this scaffold for additional biological validation.

Furthermore, the inclusion of IC_90_ parameters provides a more robust pharmacological profile, which suggests that leading hybrids, particularly **3f**, are able to achieve a parasitizing effect at significantly lower concentrations than the reference drug. This potential is critical to prevent the survival of resistant parasite populations in early infection contexts.

The strong correlation between our docking predictions and experimental results validates thioredoxin reductase as a relevant molecular target for thiazolyl–imidazole hybrids. These results provide a robust foundation for future studies on the *in vivo* efficacy of these compounds in cutaneous leishmaniasis models, with the aim of overcoming the limitations of current chemotherapy.

Overall, the evaluation in promastigotes provides a relevant initial assessment of antileishmanial activity in the early infective stage. Therefore, compounds such as **3f** should be considered promising molecules to confirm whether the observed potency and selectivity are maintained in the mammalian intracellular stage of *Leishmania* infection.

## 5. Conclusions

The present study demonstrates that the rational integration of computational design, synthetic chemistry, and biological evaluation is an effective strategy for developing novel antileishmanial agents. Initial virtual screening of a focused azole-based library identified thiazole and imidazole scaffolds as privileged structures for targeting thioredoxin reductase (4CBQ), thereby guiding the design of thiazolylimidazole hybrid systems. The successful synthesis and structural characterization of the target compounds provided a solid basis for biological validation. *In vitro* assays against *Leishmania mexicana* promastigotes revealed the pharmacochemical influence of specific functional groups, and several hybrid derivatives exhibited significantly enhanced activity compared with miltefosine, particularly compound **3f**, which showed the highest potency. Importantly, these compounds displayed a favourable cytotoxicity profile in mammalian cells, resulting in improved selectivity indices relative to the reference drug. In addition, acute toxicity studies in murine models indicated low toxicity for the most active derivatives, supporting their preliminary safety. A clear structure–activity relationship was established, highlighting the critical role of electron-withdrawing substituents at the para position of the arylthiazole moiety in enhancing antiparasitic activity. The strong agreement between docking predictions and experimental findings further supports thioredoxin reductase as a relevant molecular target. Overall, thiazolylimidazole hybrids emerge as promising early-stage scaffolds for antileishmanial drug discovery. Nevertheless, the acute toxicity data provide an initial safety-oriented criterion for prioritizing compounds such as **3f** for subsequent biological validation.

## Figures and Tables

**Table 1:** Affinity parameters of blind docking of the compounds **1a**–**f, 2** and **3a**–**f**.

Compound	Ar (*p*−4)	Binding Energy (∆G)	Inhibition Constant (nM)	* LogP
**3f**	NO_2_	−16.08	0.0019	4.33
**3e**	CN	−15.99	0.0024	4.11
**3b**	CH_3_	−12.94	0.32	4.80
**3d**	Cl	−12.06	2.31	4.94
**3a**	H	−10.7	14.33	4.27
**3c**	F	−10.54	23.01	5.03
**Miltefosine**	-	−8.21	951.45	3.44
**1f**	NO_2_	−7.69	2310	2.35
**1e**	CN	−7.49	3580	2.15
**1d**	Cl	−6.77	10890	3.07
**1b**	CH_3_	−6.62	14130	2.84
**1a**	H	−6.34	22590	2.79
**2**	-	−6.22	27892	2.54
**1c**	F	−6.15	30580	2.46

* Estimated in Molinspiration.

**Table 2:** Docking by recognition of compound series **3** on catalytic site.

Compound	Ar (*p*−4)	Binding Energy (∆G)	Inhibition Constant (nM)	LogP
**3f**	NO_2_	−15.09	0.0021	4.33
**3e**	CN	−14.99	0.0024	4.11
**3b**	CH_3_	−12.94	0.32	4.80
**3d**	Cl	−11.16	2.31	4.94
**3a**	H	−9.96	780.45	4.27
**3c**	F	−9.15	870.04	5.03
**Miltefosine**	-	−8.21	951.45	3.44

**Table 3:** Results of the synthesis of series **1**.

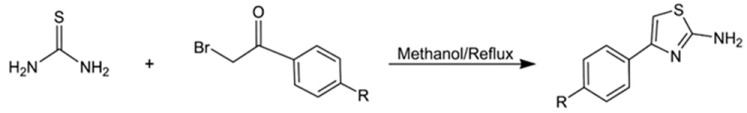			
**Compound**	**R**	**Melting Point (°C)**	**Yield (%)**
**1a**	H	148.7–150.0	90
**1b**	CH_3_	134.5–135.2	83
**1c**	F	107.7–108.5	85
**1d**	Cl	168.4–168.9	75
**1e**	CN	177.3–177.7	87
**1f**	NO_2_	159.4–159.9	72

**Table 4:** Results of the synthesis of series **3**.

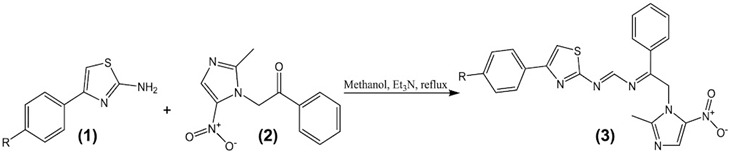			
**Compound**	**R**	**Melting Point (°C)**	**Yield (%)**
**3a**	H	257.6–258.4	49
**3b**	CH_3_	261.2–262.1	45
**3c**	F	270.5–271.2	51
**3d**	Cl	262.4–263.1	60
* **3e**	CN	Complex mix	* Nq
**3f**	NO_2_	254.4–254.8	54

*/Nq: nonquantifiable.

**Table 5:** Pharmacological response of the compounds **3a**–**f** against *L. mexicana* and toxicity in mammalian cells.

Compound	Ar (−4)	^1^ LogP	^2^ −∆G	^2^ -KI (nM)	^3^ IC_50_ (µM)	^3^* IC_90_ (µM)	^4^ MIC (µM)	^5^ CC_50_ (µM)	SI
* **3f**	NO_2_	4.33	16.08	0.0019	22.41	201.69	0.42	2000.2	89.3
* **3b**	CH_3_	4.35	15.99	0.32	96.03	864.27	1.82	2016.8	21
* **3d**	Cl	3.62	12.06	2.31	100.39	903.51	0.87	2231.6	22.2
**1f**	NO_2_	2.35	7.69	2310	119.50	1075.50	1.72	4072.6	18.6
**MTF**	-	2.68	8.21	951.45	132.42	1191.78	3.75	3556.3	26.9
**1d**	Cl	3.07	6.77	10,890	347.99	3131.91	7.26	4425.2	12.7
**1b**	CH_3_	2.84	6.62	14,130	406.63	3659.67	8.04	4834.8	11.9
**3a**	H	3.62	10.7	14.33	430.93	3878.37	124	2120	4.9
**3c**	F	4.25	10.54	23.01	644.25	5798.25	29.66	1893.2	2.9
**1a**	H	2.39	6.34	22,590	1446.43	13,017.87	70.93	5100	3.5
**2**	H	1.68	6.22	27,892	1661.45	14,953.05	203.88	3224.2	1.9
**1c**	F	2.56	6.15	30,580	2141.54	19,273.86	64.34	4664	2.2

* Indicates a statistically significant difference compared to Miltefosine according to Dunnett’s test (*p* < 0.05). ^1^ Calculated in Molinspiration; ^2^ Obtained in Autodock Tools; ^3^ Calculated by PROBIT model; ^3^* IC_90_ values were estimated based on the experimental IC_50_ results assuming a standard Hill slope (H = 1.0); ^4^ MIC: Minimum inhibitory concentration; endpoint in the resazurin/Alamar Blue assay; ^5^ Cytotoxicity in Vero Cells.

**Table 6:** Acute toxicity (lethal dose) of the series **3** compounds.

Compound	R	LD_50_ (mg/kg)
**3a**	H	>2000
**3b**	CH_3_	>2000
**3c**	F	500–1000
**3d**	Cl	500
**3f**	NO_2_	>2000

## Data Availability

The data presented in this study are available in the article and its Supplementary Material. Further inquiries can be directed to the corresponding author.

## Supplementary Materials

The following supporting information can be downloaded at: https://www.mdpi.com/article/10.3390/pathogens15050544/s1, Figure S1: IR spectrum of compound 1f; Figure S2: ^1^H NMR spectrum of compound 1f; Figure S3: ^13^C NMR spectrum of compound 1; Figure S4: ^1^H-^1^H COSY spectrum of compound 1f; Figure S5: ^1^H–^13^C HMBC spectrum of compound 1f; Figure S6: IR spectrum of compound 2; Figure S7: ^1^H NMR spectrum of compound 2; Figure S8: ^13^C NMR spectrum of compound 2; Figure S9: ^1^H-^1^H COSY spectrum of compound 2; Figure S10: ^1^H–^13^C HMBC spectrum of compound 2; Figure S11: IR spectrum of compound 3a; Figure S12: ^1^H NMR spectrum of compound 3a; Figure S13: ^13^C NMR spectrum of compound 3a; Figure S14: ^1^H-^1^H COSY spectrum of compound 3a; Figure S15: ^1^H–^13^C HMBC spectrum of compound 3a; Figure S16: IR spectrum of compound 3b; Figure S17: ^1^H NMR spectrum of compound 3b; Figure S18: ^13^C NMR spectrum of compound 3b; Figure S19: ^1^H-^1^H COSY spectrum of compound 3b; Figure S20: ^1^H–^13^C HMBC spectrum of compound 3b; Figure S21: IR spectrum of compound 3c; Figure S22: ^1^H NMR spectrum of compound 3c; Figure S23: ^13^C NMR spectrum of compound 3c; Figure S24: ^1^H-^1^H COSY spectrum of compound 3c; Figure S25: ^1^H–^13^C HMBC spectrum of compound 3c; Figure S26: IR spectrum of compound 3d; Figure S27: ^1^H NMR spectrum of compound 3d; Figure S28: ^13^C NMR spectrum of compound 3d; Figure S29: ^1^H-^1^H COSY spectrum of compound 3d; Figure S30: ^1^H–^13^C HMBC spectrum of compound 3d; Figure S31: IR spectrum of compound 3f; Figure S32: ^1^H NMR spectrum of compound 3f; Figure S33: ^13^C NMR spectrum of compound 3f; Figure S34: ^1^H-^1^H COSY spectrum of compound 3f; Figure S35: ^1^H–^13^C HMBC spectrum of compound 3f.

## Abbreviations

The following abbreviations are used in this manuscript:

ATCC	American Type Culture Collection
ATR	Attenuated Total Reflectance
b.w.	Body Weight
CC_50_	Cytotoxic concentration 50%
CEI	Ethics and Research Committee
CH_3_	Methyl group
Cl	Chloro group
CL	Cutaneous Leishmaniasis
CN	Cyano group
COSY	Correlation System Spectroscopy
DMSO	Dimethyl Sulfoxide
F	Fluoro group
FBS	Fetal Bovine Serum
∆G	Gibbs Free Energy (binding free energy)
H	Hydrogen
HMBC	Heteronuclear Multiple Bond Correlation
IC_50_	Inhibitory concentration 50%
IC_90_	Inhibitory concentration 90%
IR	Infrared Spectra
Ka/∆G	Binding energy
KI	Inhibitory Constant
LGA	Lamarckian Genetic Algorithm
LD	Lethal dose
LD_50_	Lethal dose 50%
LogP	Partition coefficient
MIC	Minimum inhibitory concentration
MTF	Miltefosine
NO_2_	Nitro group
OECD	Organization for Economic Co-operation and Development (Test 423)
PDB	Protein Data Bank
Ph	Phenyl group
PROBIT	Probability Unit Regression Method
RPMI	Roswell Park Memorial Institute Medium
TLC	Thin Layer Chromatography
TR	Thioredoxin reductase
TryR	Trypanothione reductase
