# Supplementary material for: Novel Thiazolylimidazole Hybrids as Promising Antileishmanial Agents: Rational Design and Biological Evaluation
Source: Pathogens. 2026 May 18;15(5):544. doi: 10.3390/pathogens15050544 (PMC13209422; doi:10.3390/pathogens15050544)
Supplement: Supplementary file 1 [file pathogens-15-00544-s001.zip › pathogens-4289831-supplementary.pdf]

Supplementary Information

Novel Thiazolyl—Imidazole Hybrids as Promising Antileishmanial Agents: Rational Design and Biological Evaluation

Cristoper Ramírez-Sandoval, María Elena Campos-Aldrete, María Estela Meléndez-Camargo

TABLE OF CONTENTS

Figure S1. IR spectrum of compound 1f.
Figure S2. ^{1}H NMR spectrum of compound 1f.
Figure S3. ^{13}C NMR spectrum of compound 1f.
Figure S4. ^{1}H-^{1}H COSY spectrum of compound 1f.
Figure S5. ^{1}H–^{13}C HMBC spectrum of compound 1f.
Figure S6. IR spectrum of compound 2.
Figure S7. ^{1}H NMR spectrum of compound 2.
Figure S8. ^{13}C NMR spectrum of compound 2.
Figure S9. ^{1}H-^{1}H COSY spectrum of compound 2.
Figure S10. ^{1}H–^{13}C HMBC spectrum of compound 2.
Figure S11. IR spectrum of compound 3a.
Figure S12. ^{1}H NMR spectrum of compound 3a.
Figure S13. ^{13}C NMR spectrum of compound 3a.
Figure S14. ^{1}H-^{1}H COSY spectrum of compound 3a.
Figure S15. ^{1}H–^{13}C HMBC spectrum of compound 3a.
Figure S16. IR spectrum of compound 3b.
Figure S17. ^{1}H NMR spectrum of compound 3b.
Figure S18. ^{13}C NMR spectrum of compound 3b.
Figure S19. ^{1}H-^{1}H COSY spectrum of compound 3b.
Figure S20. ^{1}H–^{13}C HMBC spectrum of compound 3b.
Figure S21. IR spectrum of compound 3c.
Figure S22. ^{1}H NMR spectrum of compound 3c.
Figure S23. ^{13}C NMR spectrum of compound 3c.
Figure S24. ^{1}H-^{1}H COSY spectrum of compound 3c.
Figure S25. ^{1}H–^{13}C HMBC spectrum of compound 3c.
Figure S26. IR spectrum of compound 3d.
Figure S27. ^{1}H NMR spectrum of compound 3d.
Figure S28. ^{13}C NMR spectrum of compound 3d.
Figure S29. ^{1}H-^{1}H COSY spectrum of compound 3d.
Figure S30. ^{1}H–^{13}C HMBC spectrum of compound 3d.
Figure S31. IR spectrum of compound 3f.
Figure S32. ^{1}H NMR spectrum of compound 3f.
Figure S33. ^{13}C NMR spectrum of compound 3f.
Figure S34. ^{1}H-^{1}H COSY spectrum of compound 3f.
Figure S35. ^{1}H–^{13}C HMBC spectrum of compound 3f.

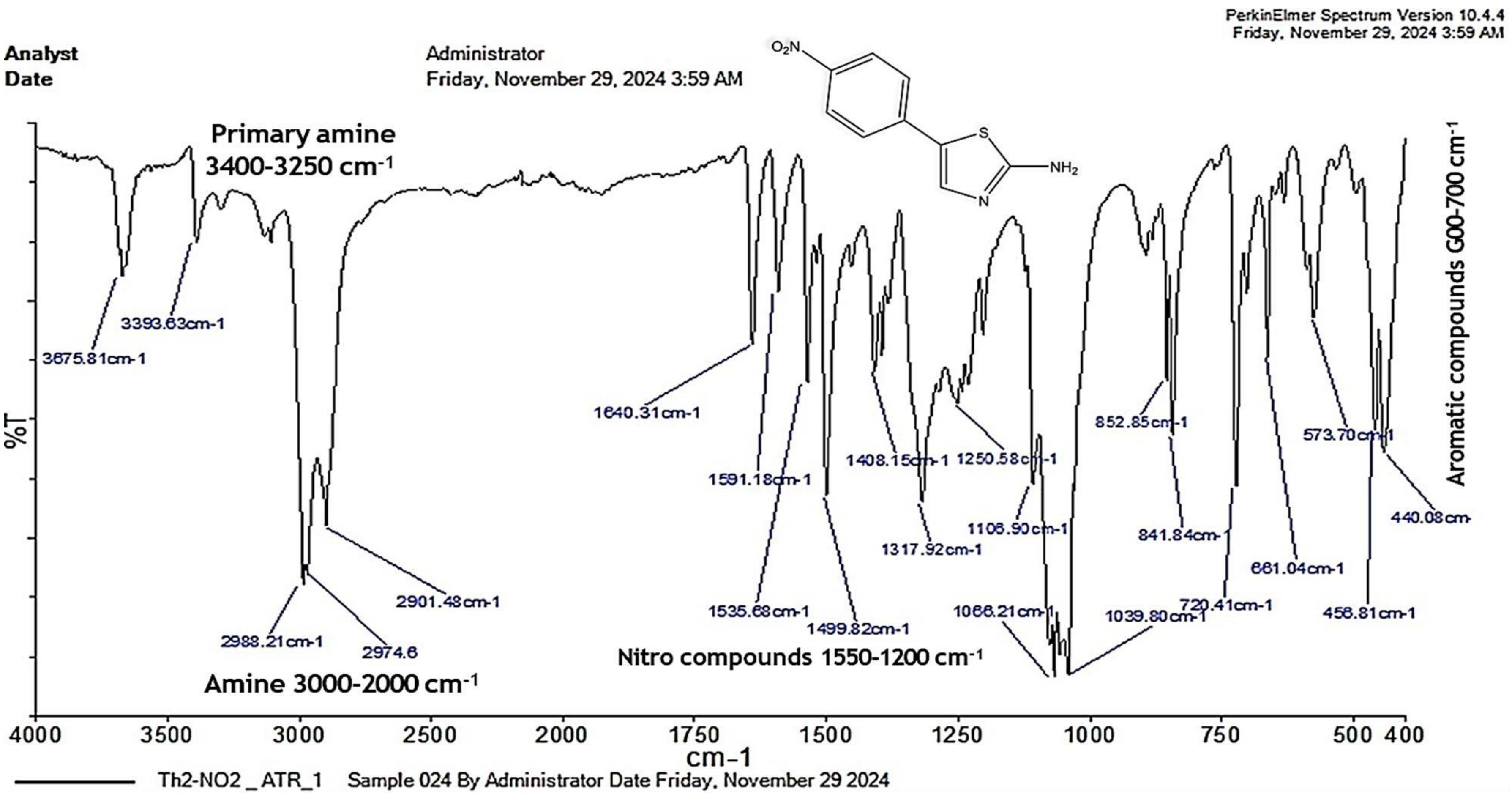

Figure S1. IR spectrum of compound 1f.

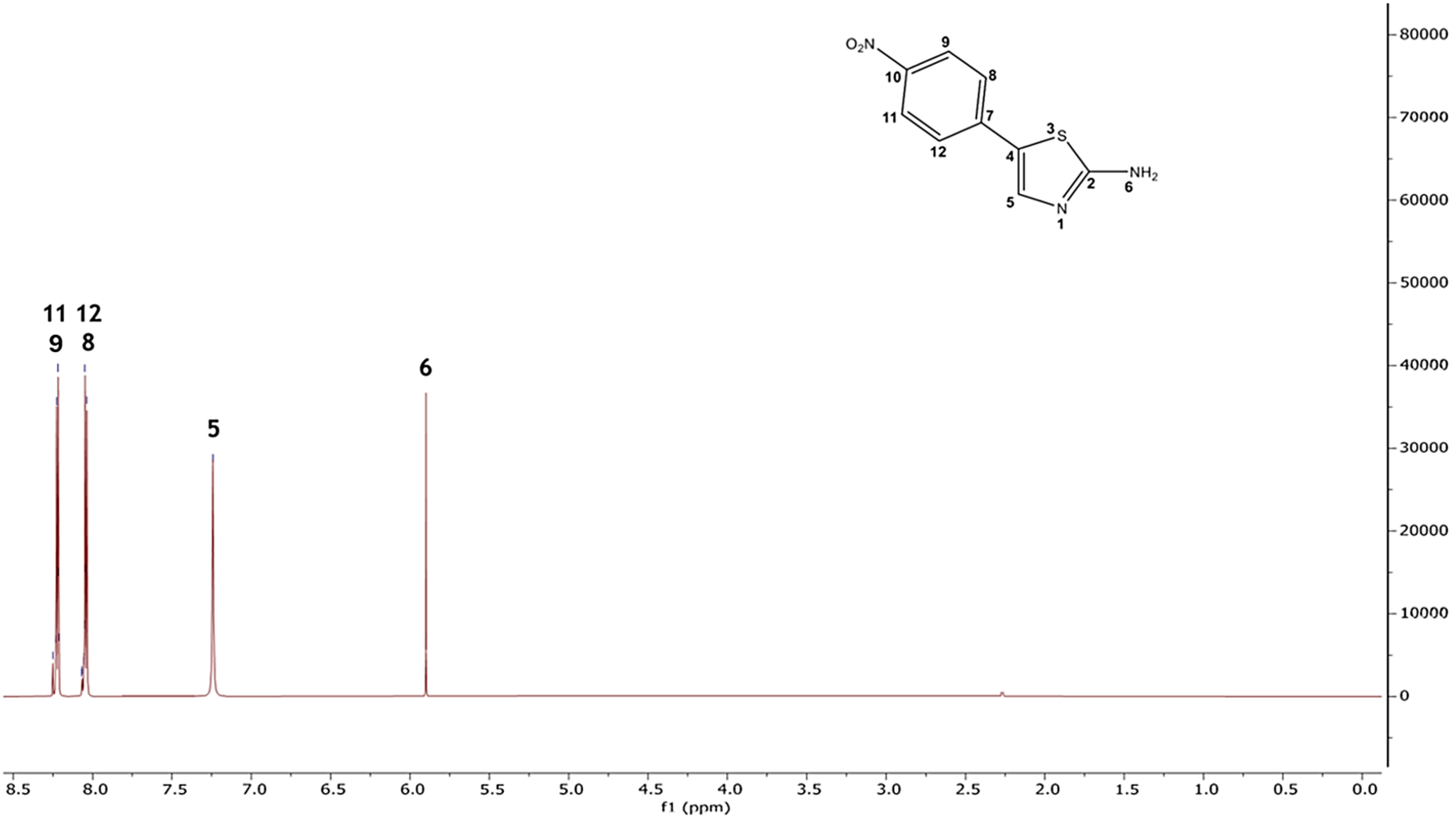

Figure S2. ^{1}H NMR spectrum of compound 1f (750 MHz, DMSO-d6).

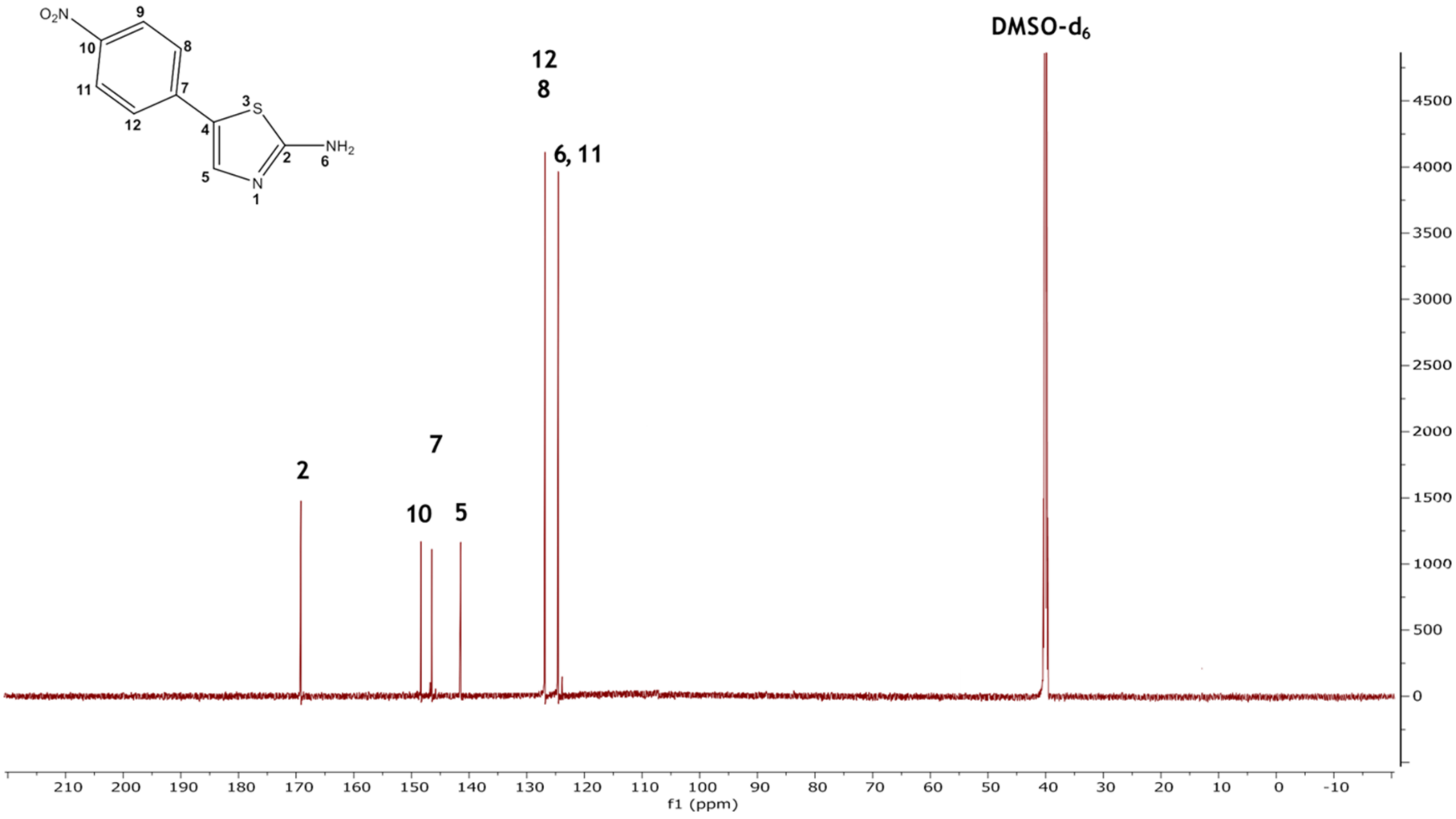

Figure S3. ^{13}C NMR spectrum of compound 1f (189 MHz, DMSO-d6).

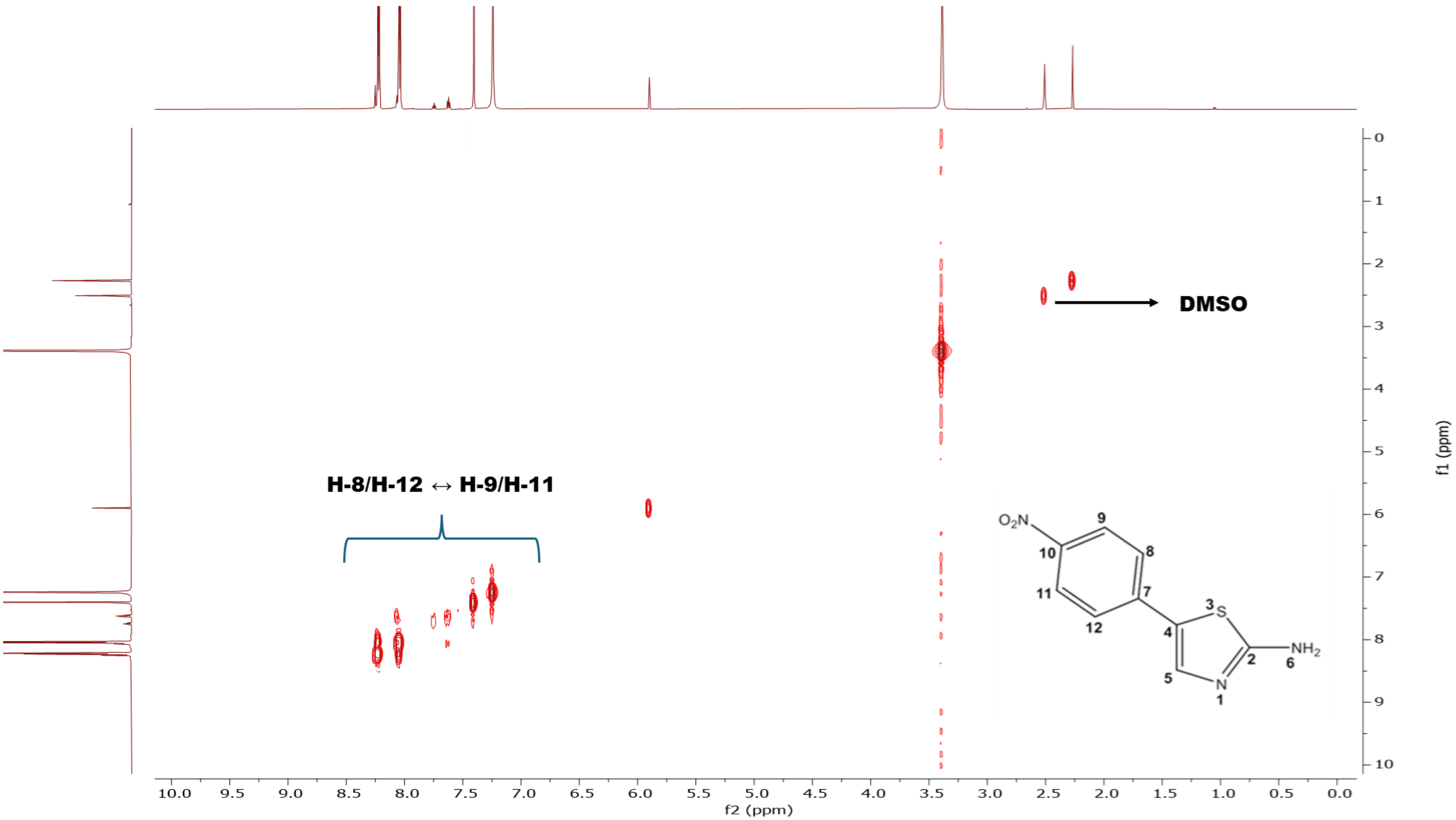

Figure S4. ^1H-^1H COSY spectrum of compound 1f (750 MHz, DMSO-d6)

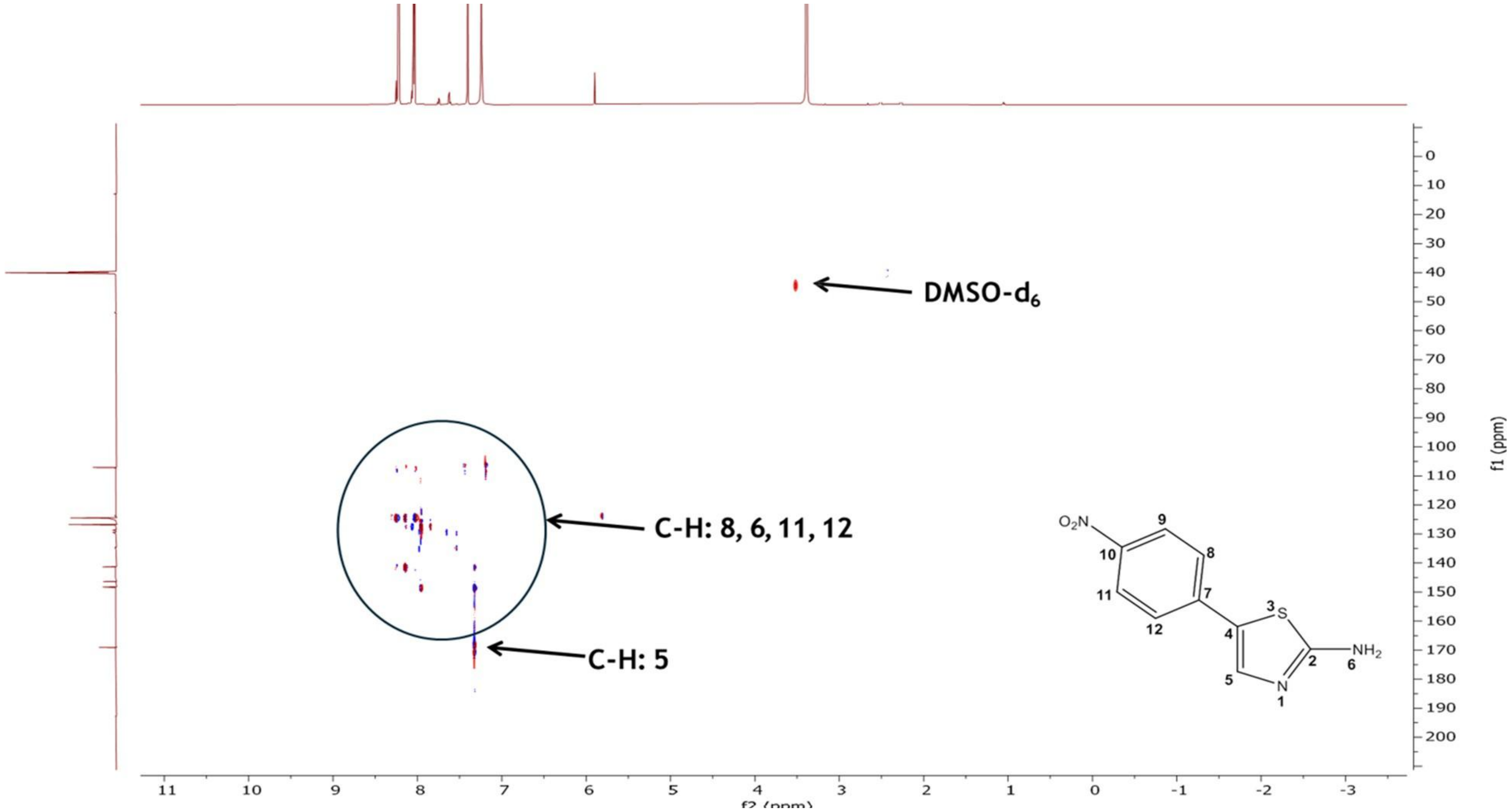

Figure S5. ^{1}H–^{13}C HMBC spectrum of compound 1f (750 MHz for ^{1}H, 189 MHz for ^{13}C, DMSO-d6).

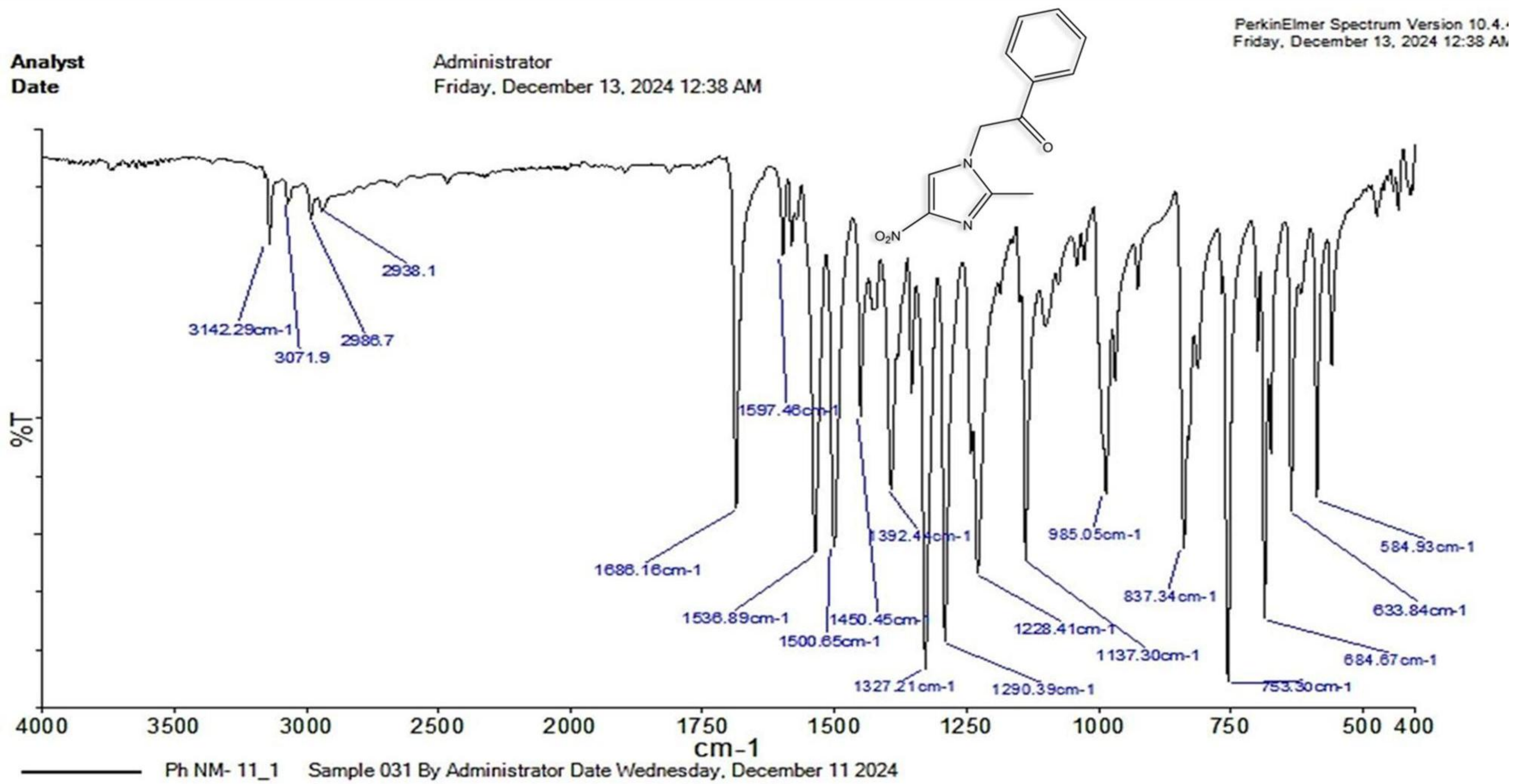

Figure S6. IR spectrum of compound 2.

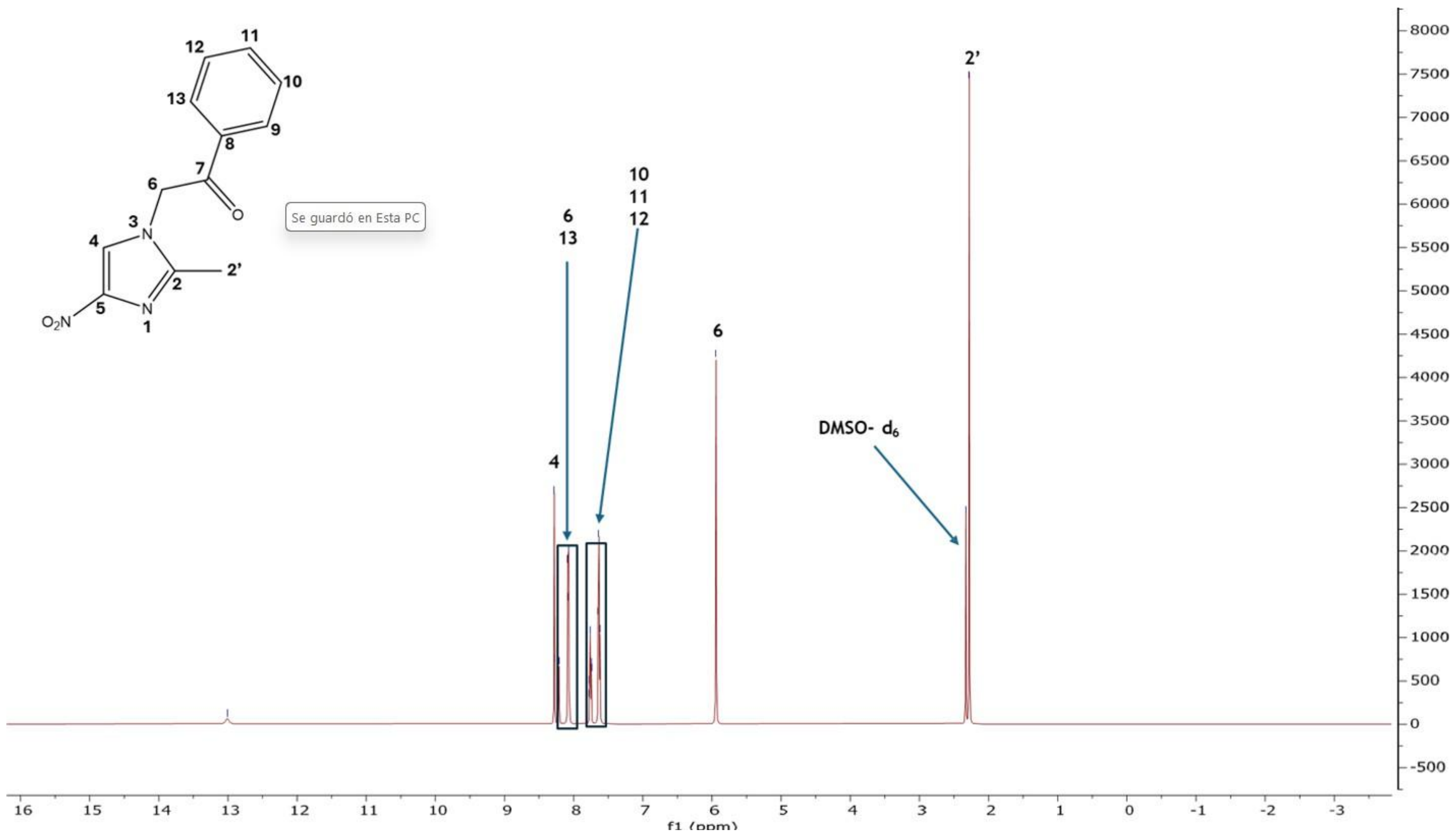

Figure S7. ^{1}H NMR spectrum of compound 2 (600 MHz, DMSO-d6)

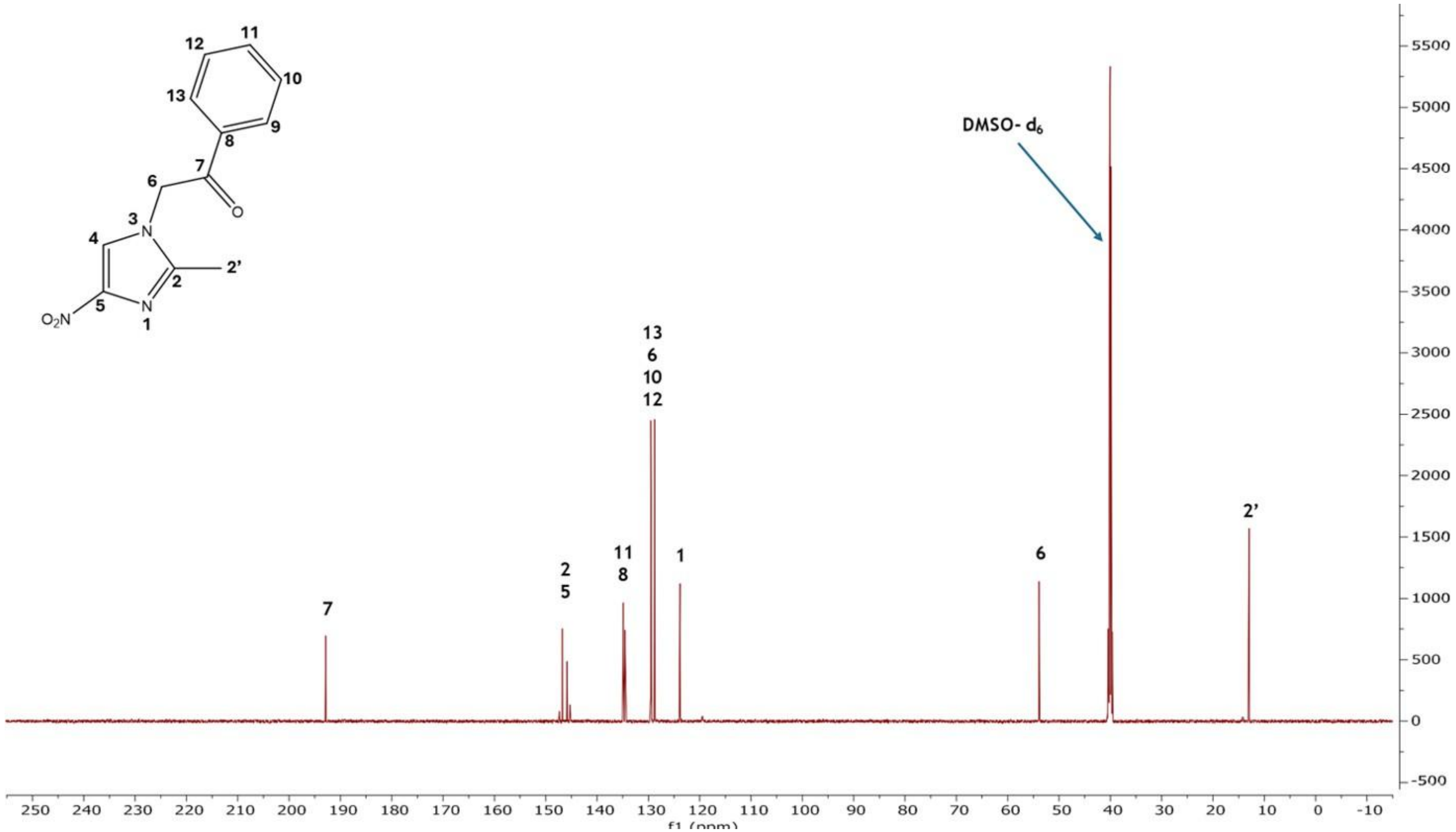

Figure S8. ^{13}C NMR spectrum of compound 2 (151 MHz, DMSO-d6).

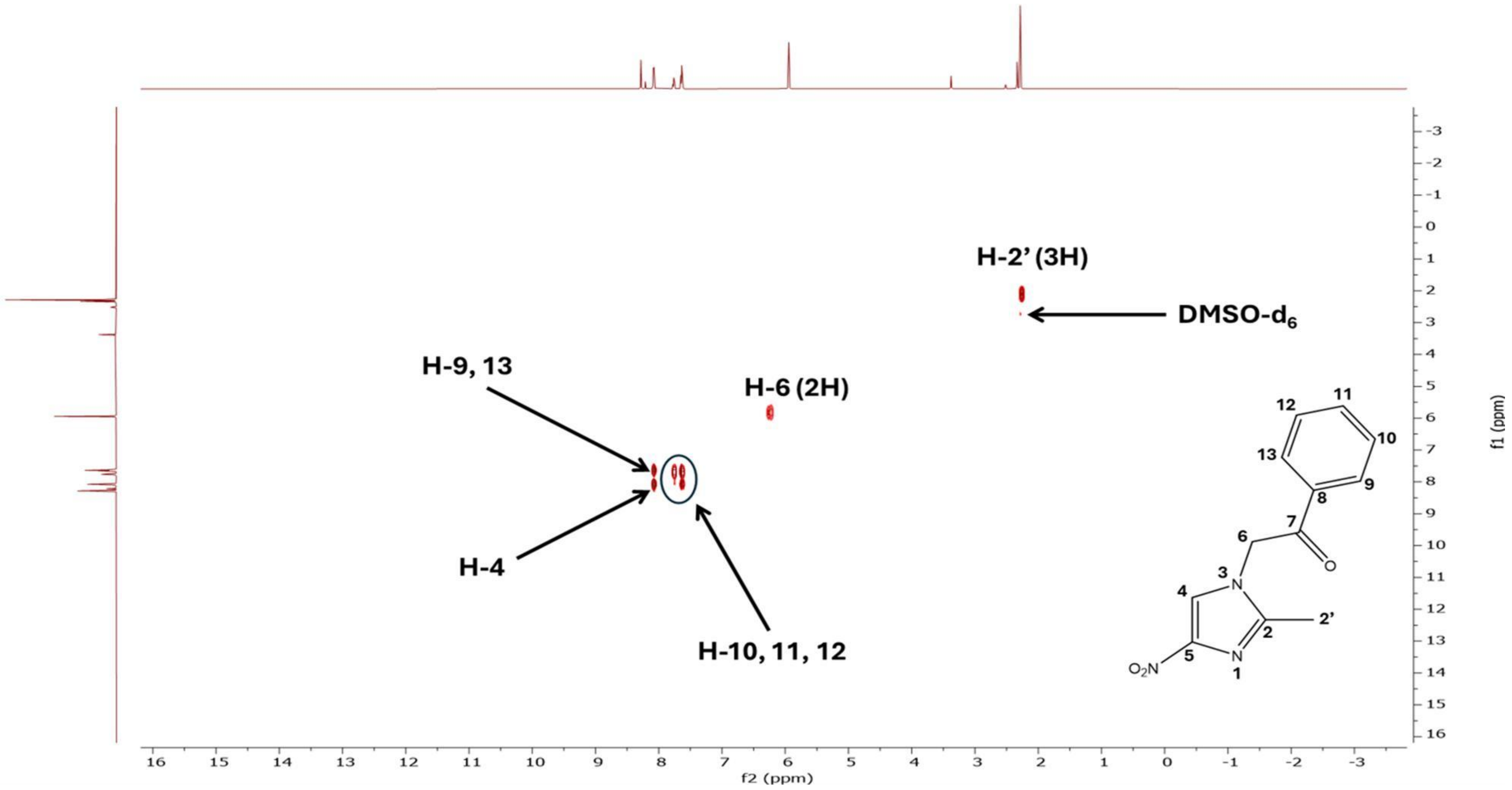

Figure S9. ^{1}H-^{1}H COSY spectrum of compound 2 (600 MHz, DMSO-d6)

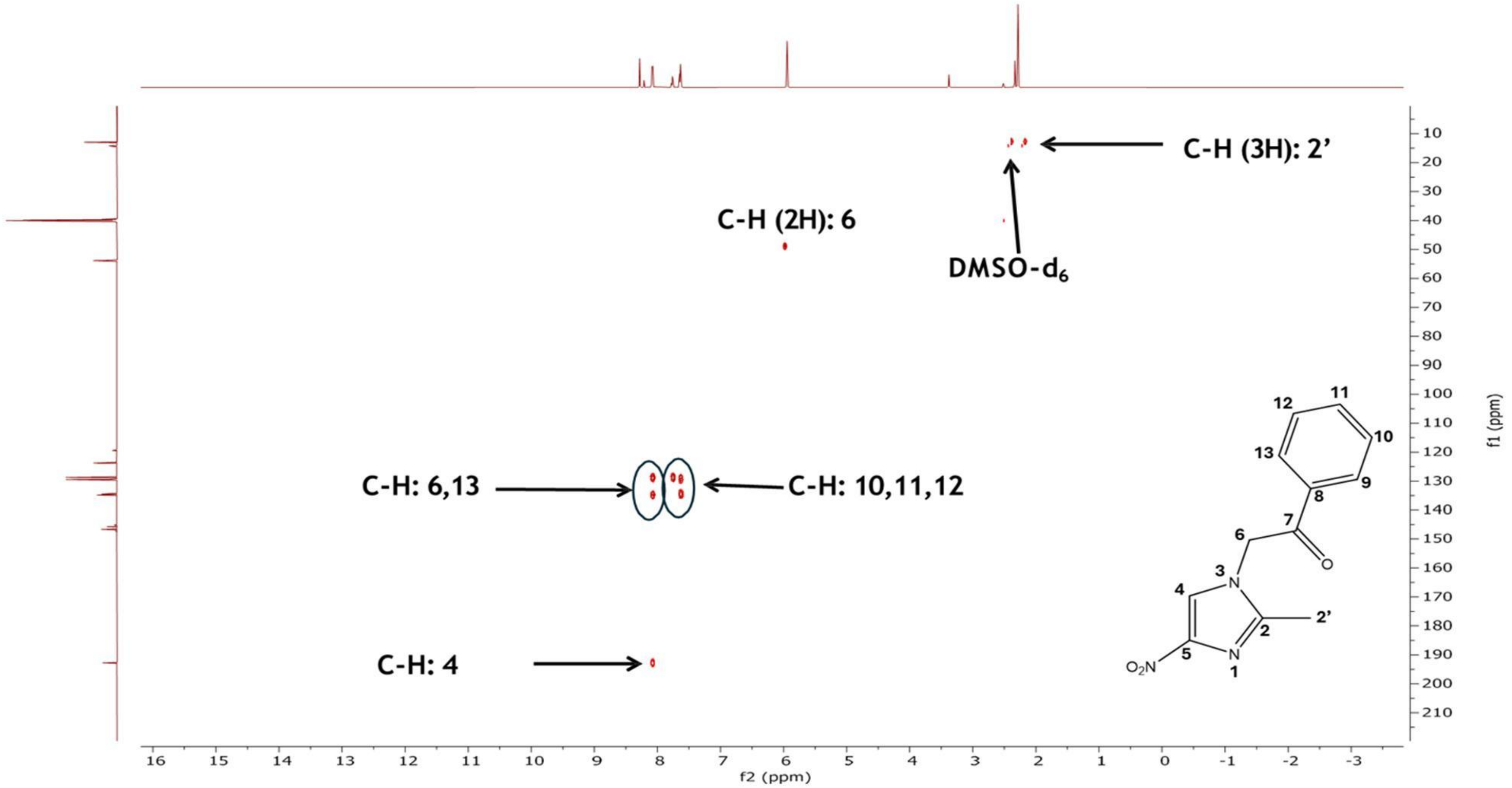

Figure S10. ^{1}H–^{13}C HMBC spectrum of compound 2 (600 MHz for ^{1}H, 151 MHz for ^{13}C, DMSO-d6).

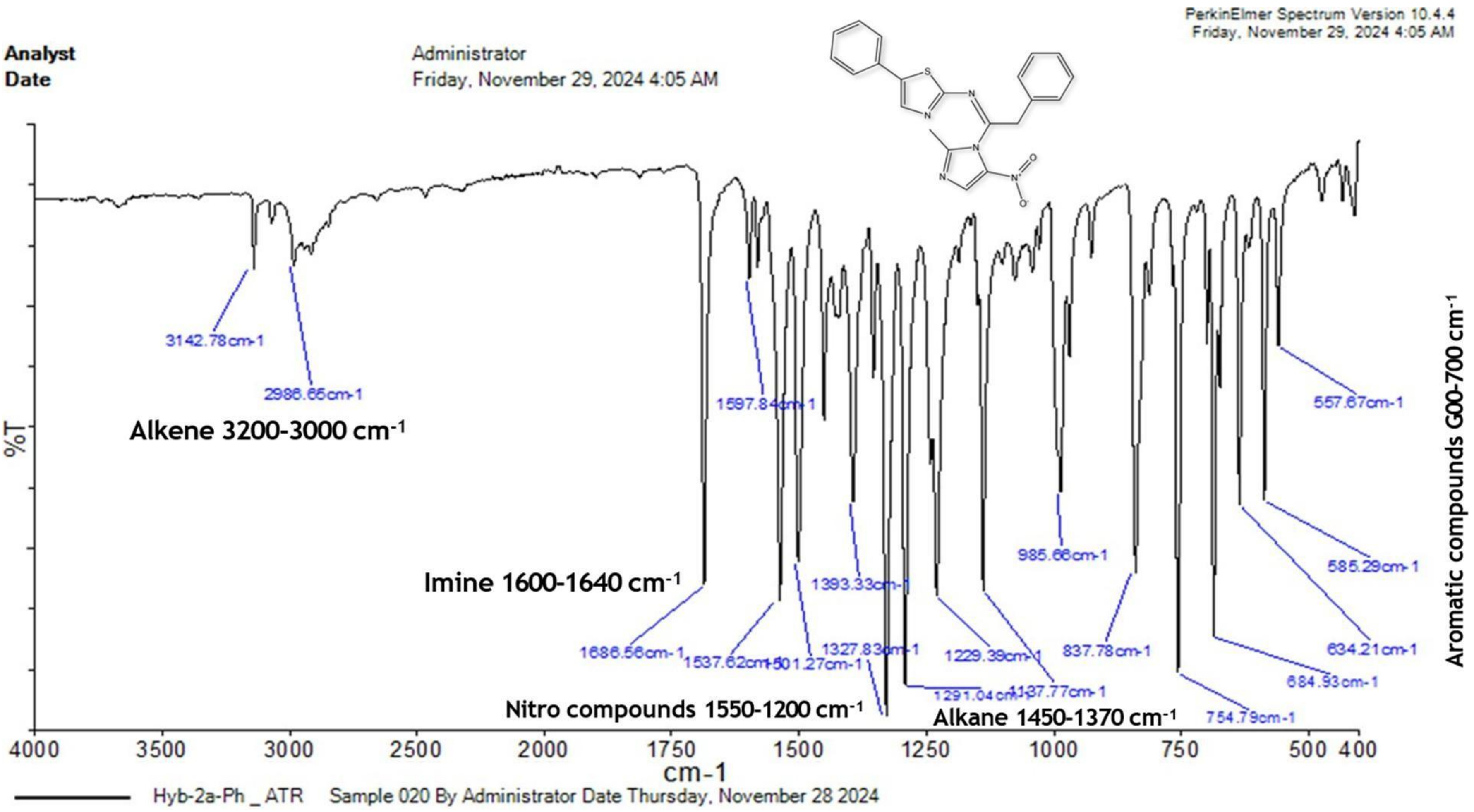

Figure S11. IR spectrum of compound 3a.

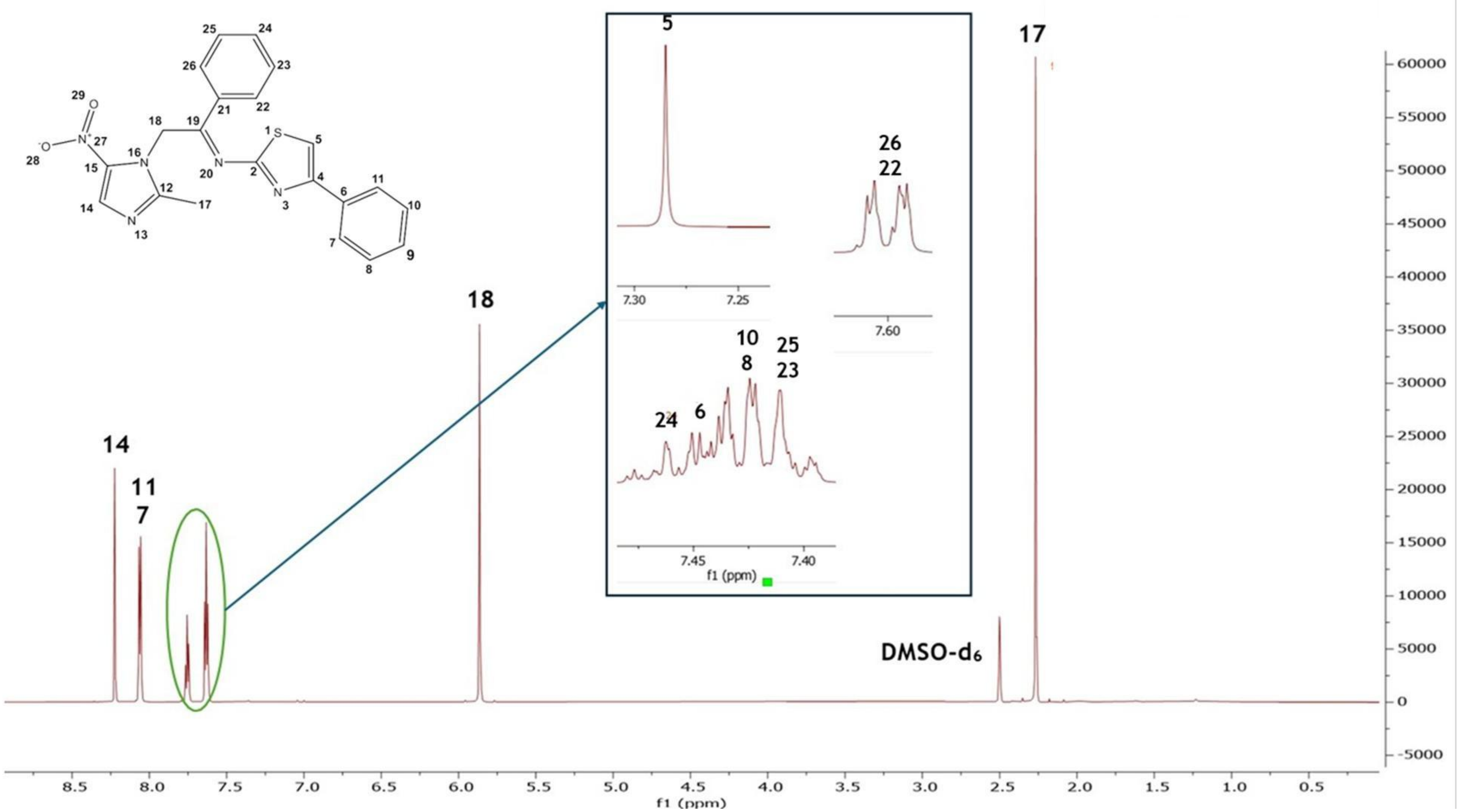

Figure S12. ^{1}H NMR spectrum of compound 3a (750 MHz, DMSO-d6).

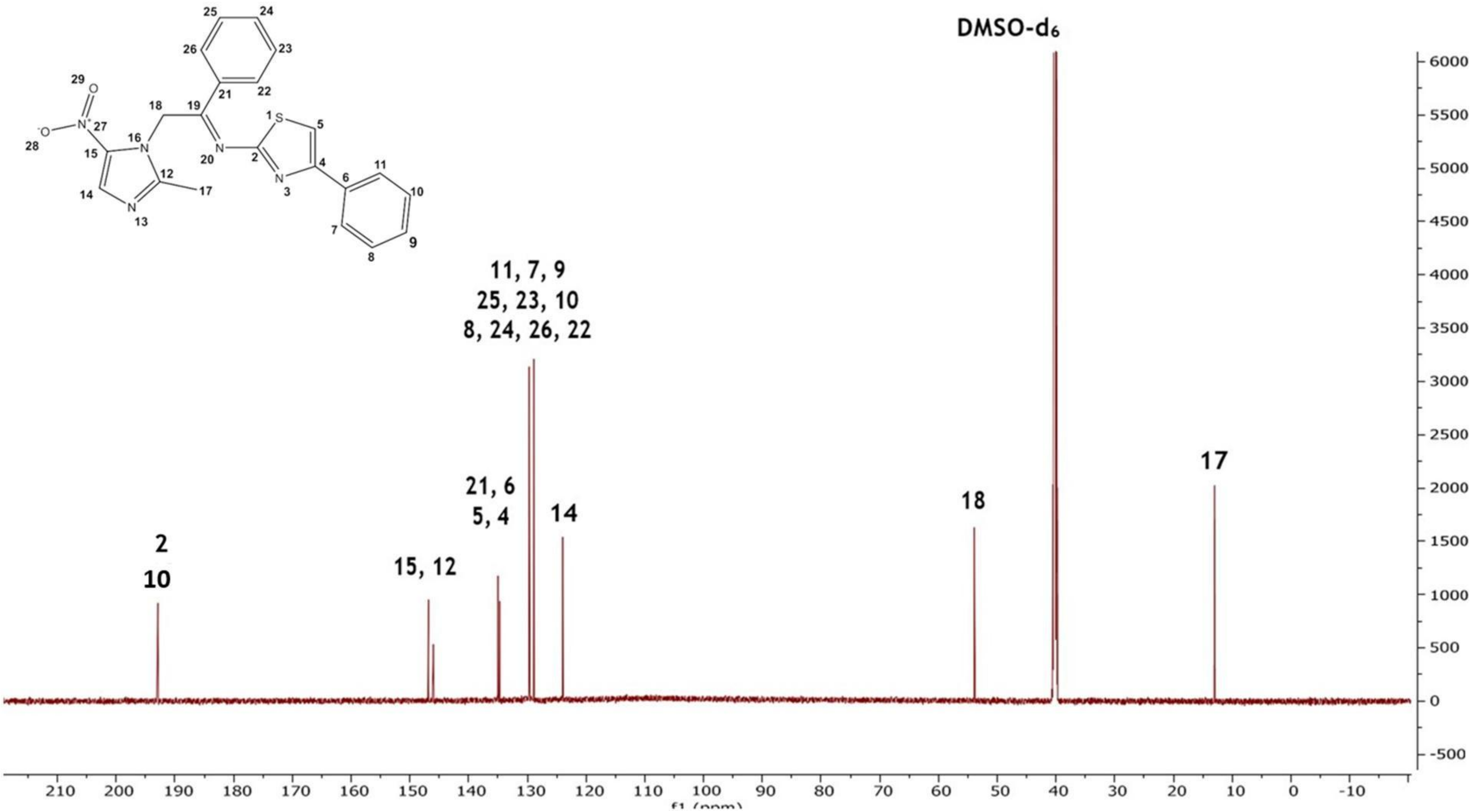

Figure S13. ^{13}C NMR spectrum of compound 3a (189 MHz, DMSO-d6).

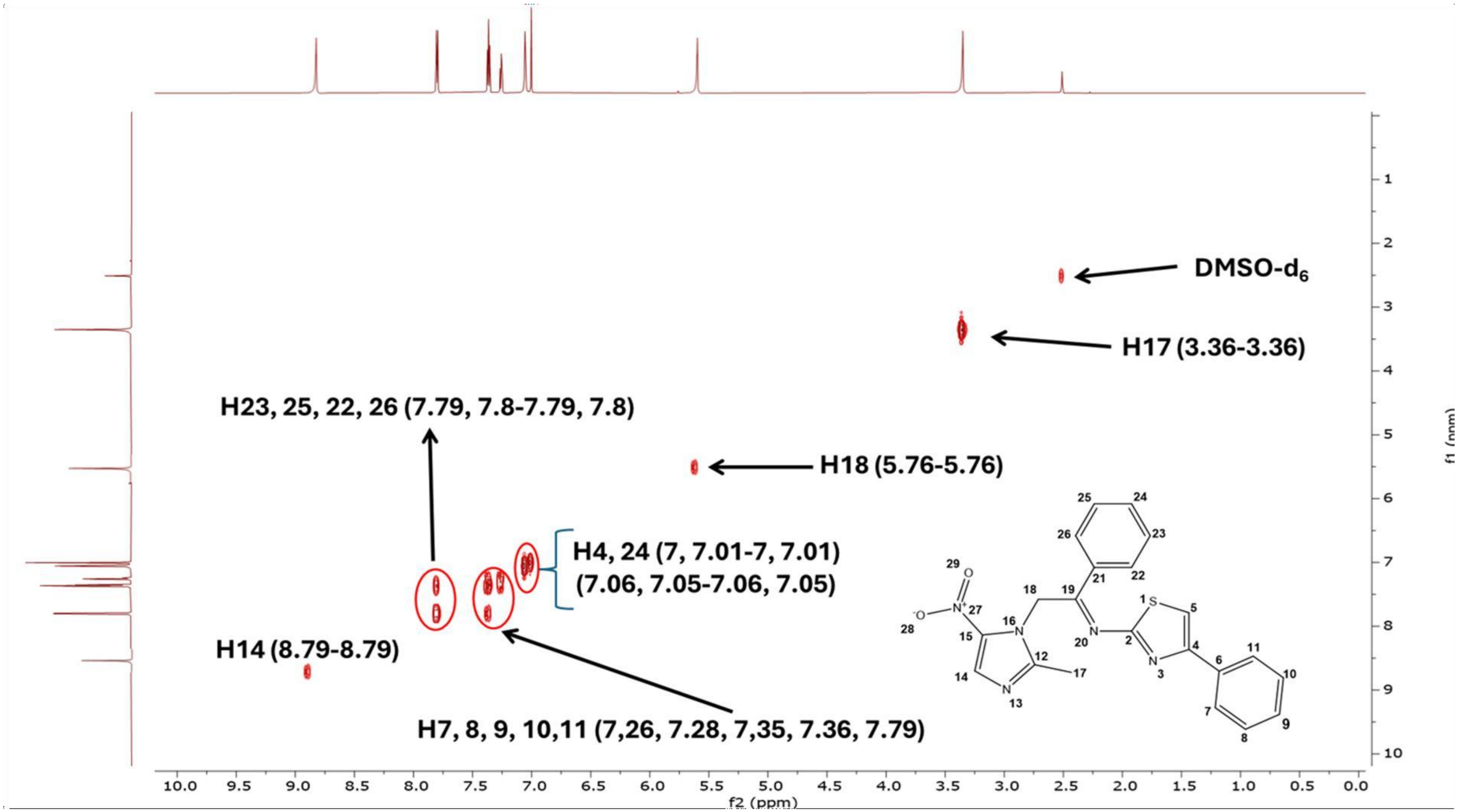

Figure S14. ^{1}H-^{1}H COSY spectrum of compound 3a (750 MHz, DMSO-d6).

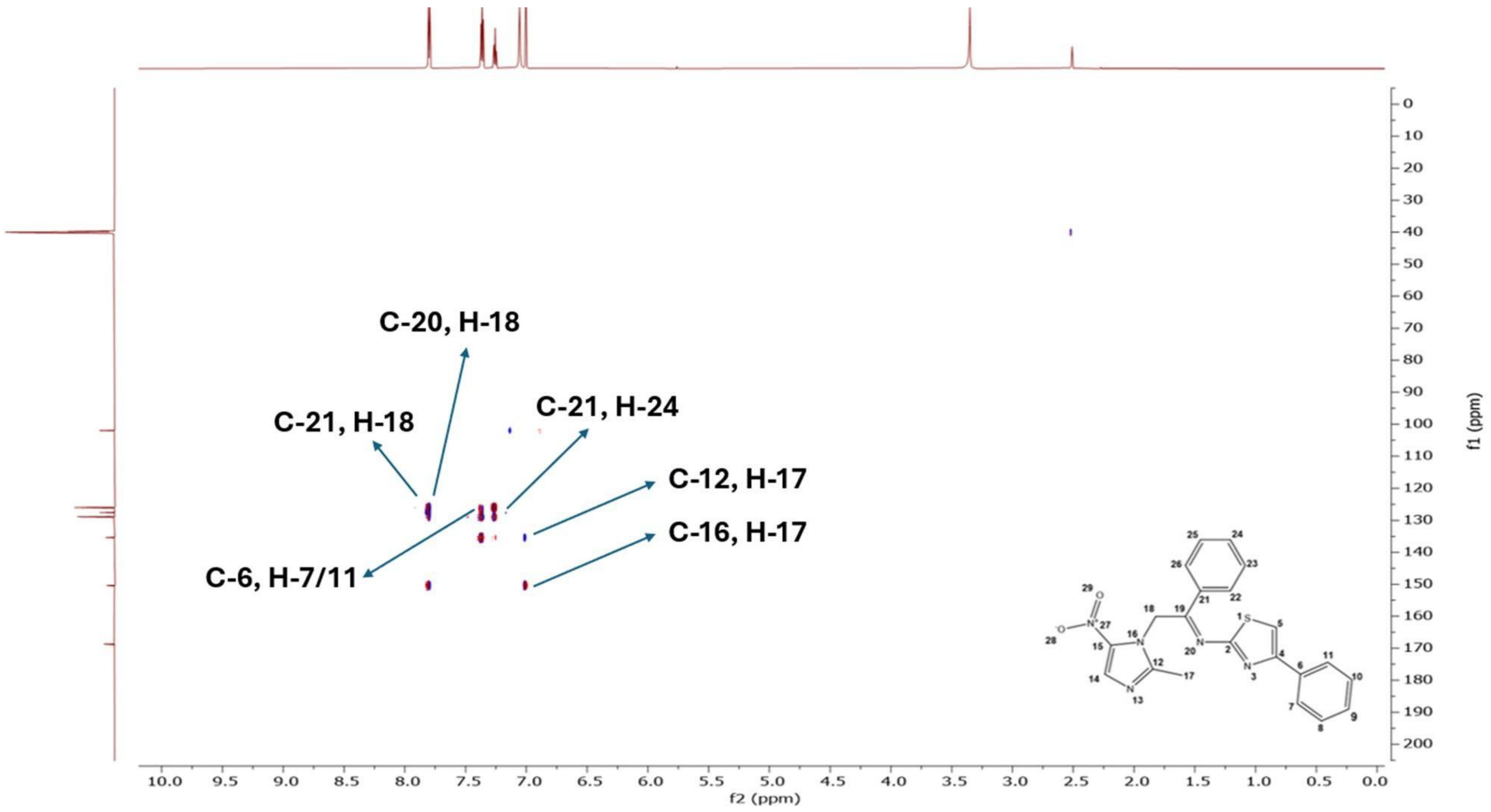

Figure S15. ^{1}H–^{13}C HMBC spectrum of compound 3a (750 MHz for ^{1}H, 189 MHz for ^{13}C, DMSO-d6).

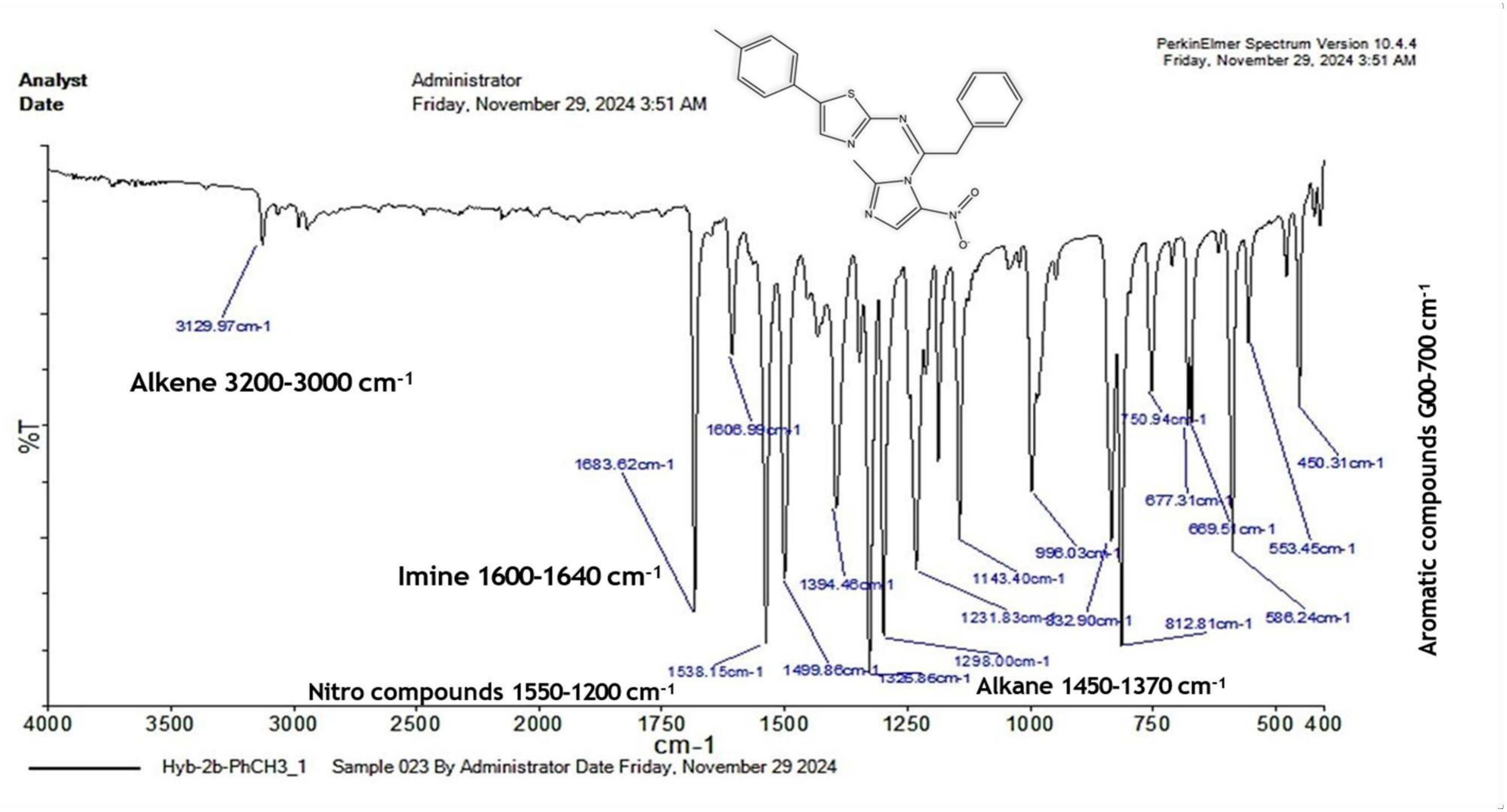

Figure S16. IR spectrum of compound 3b.

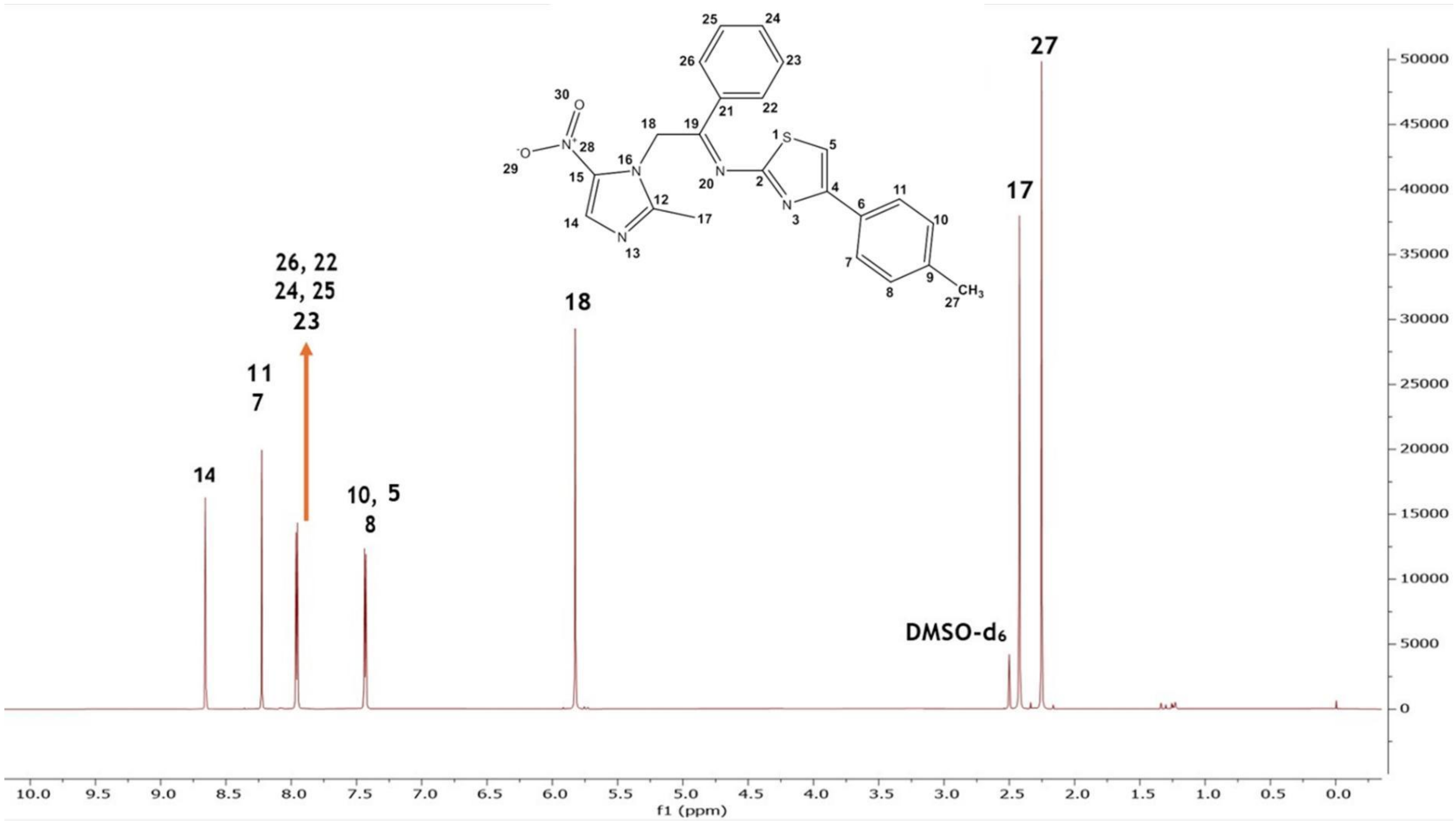

Figure S17. ^{1}H NMR spectrum of compound 3b (750 MHz, DMSO-d6)

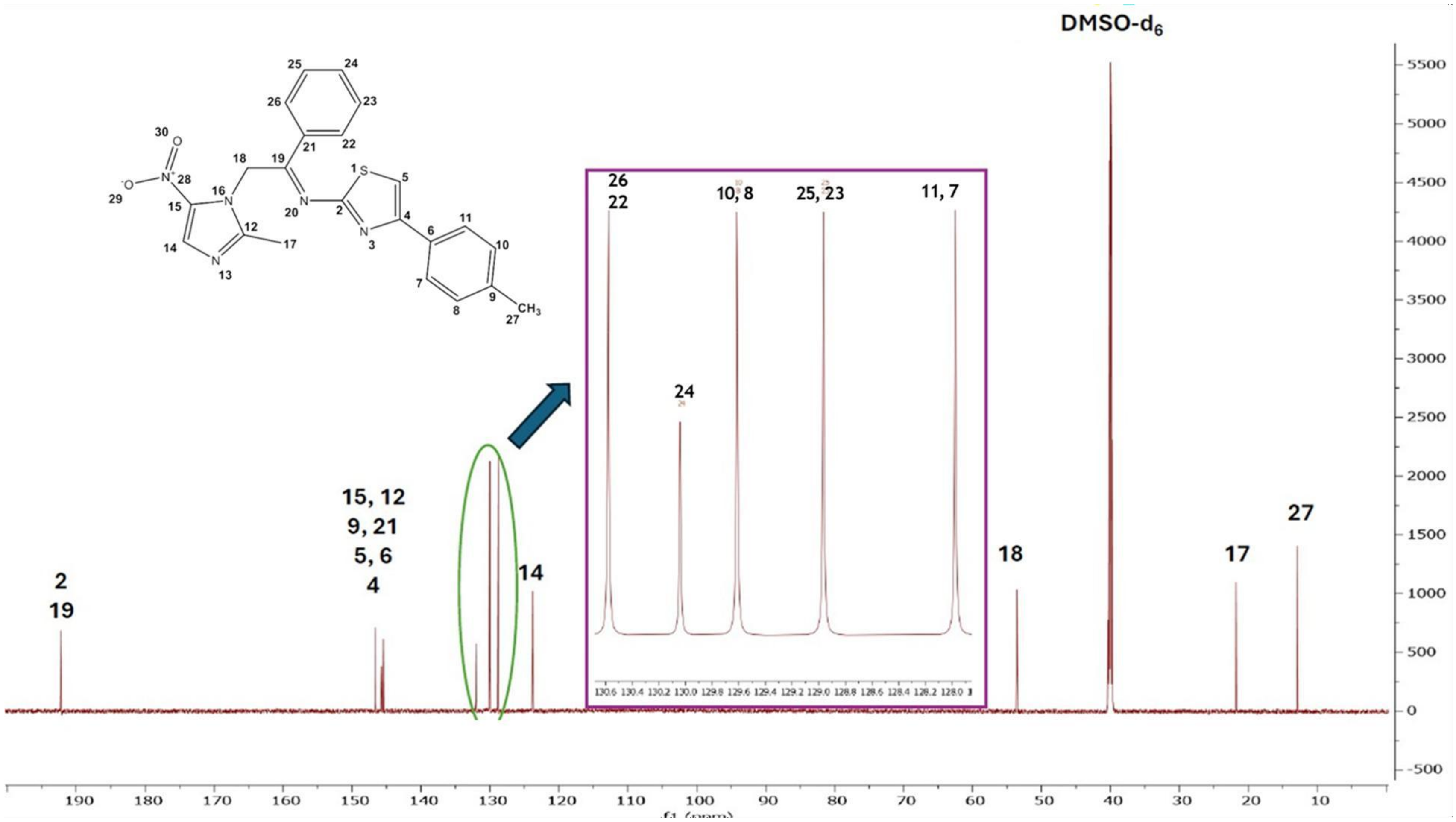

Figure S18. ^{13}C NMR spectrum of compound 3b (189 MHz, DMSO-d6).

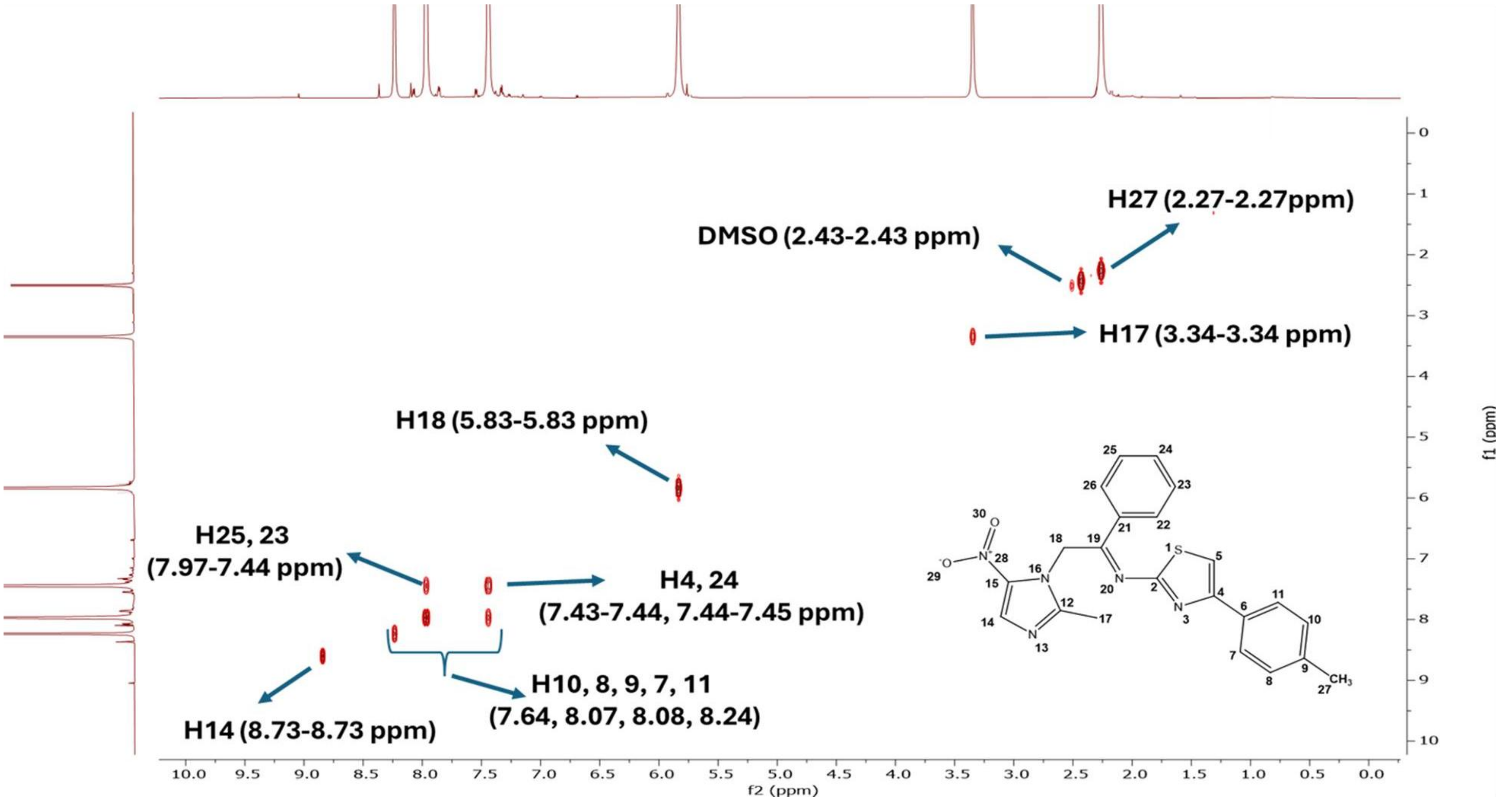

Figure S19. ^1H-^1H COSY spectrum of compound 3b (750 MHz, DMSO-d6).

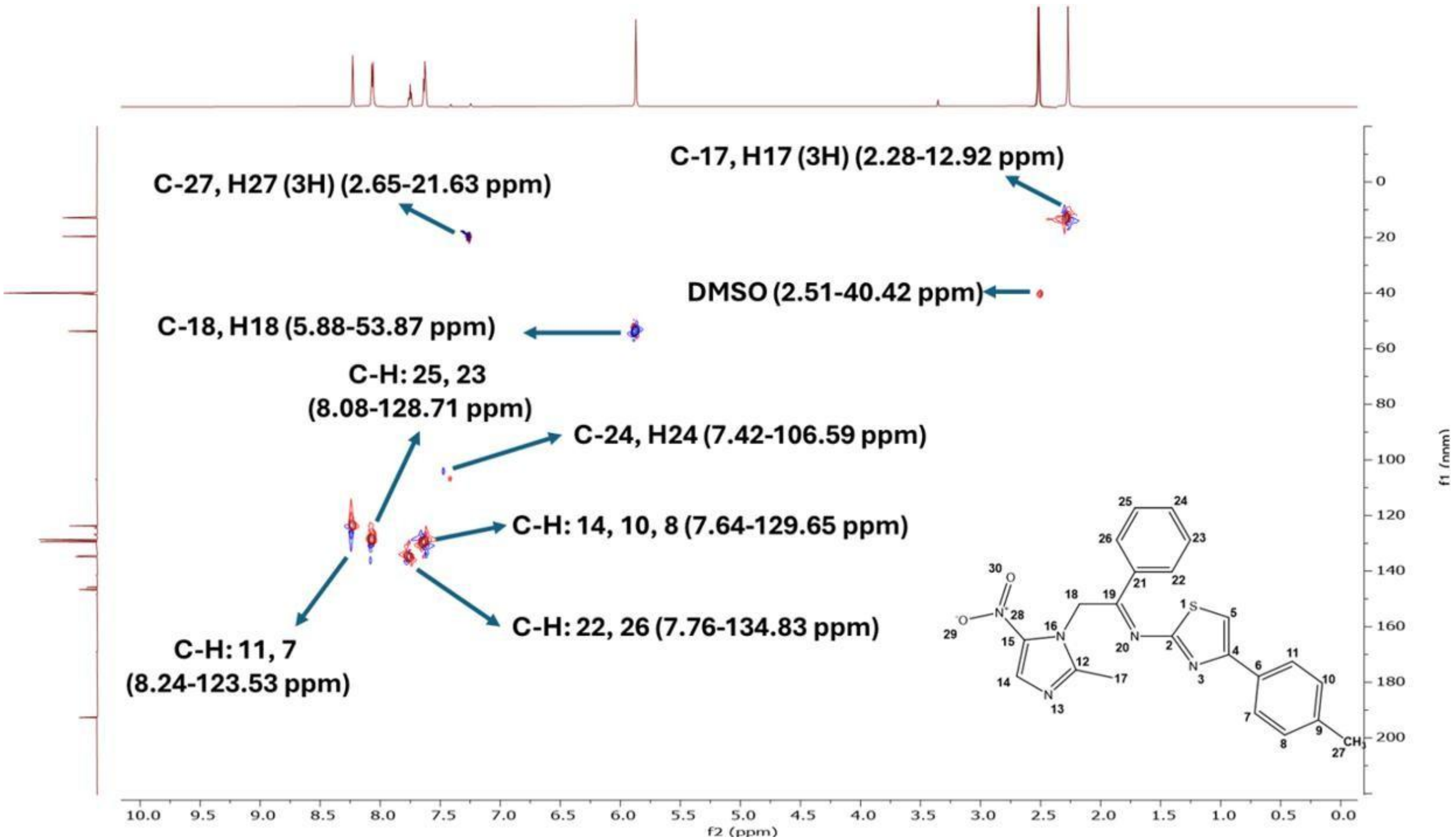

Figure S20. ^1H–^{13}C HMBC spectrum of compound 3b (750 MHz for ^1H, 189 MHz for ^{13}C, DMSO-d6).

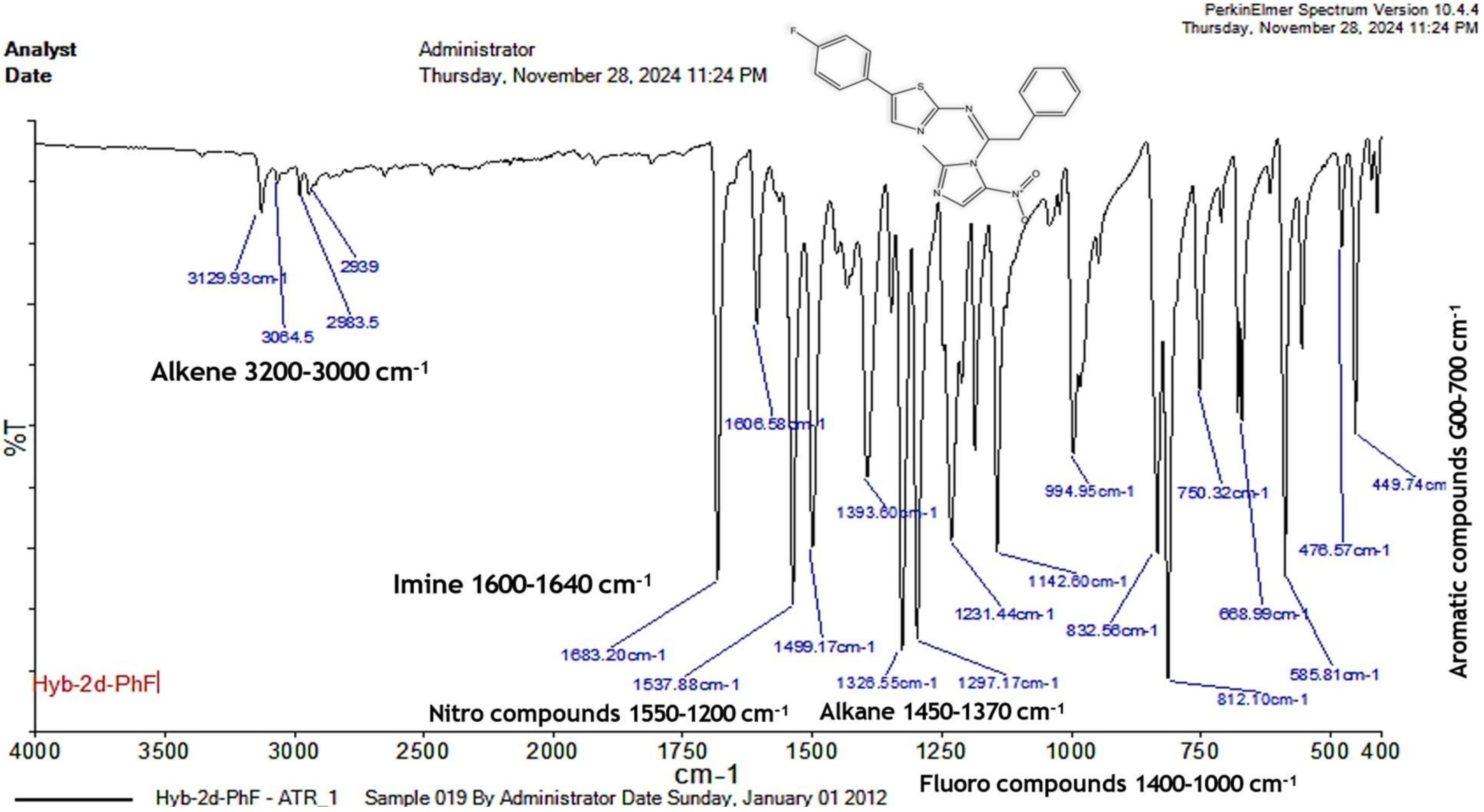

Figure S21. IR spectrum of compound 3c.

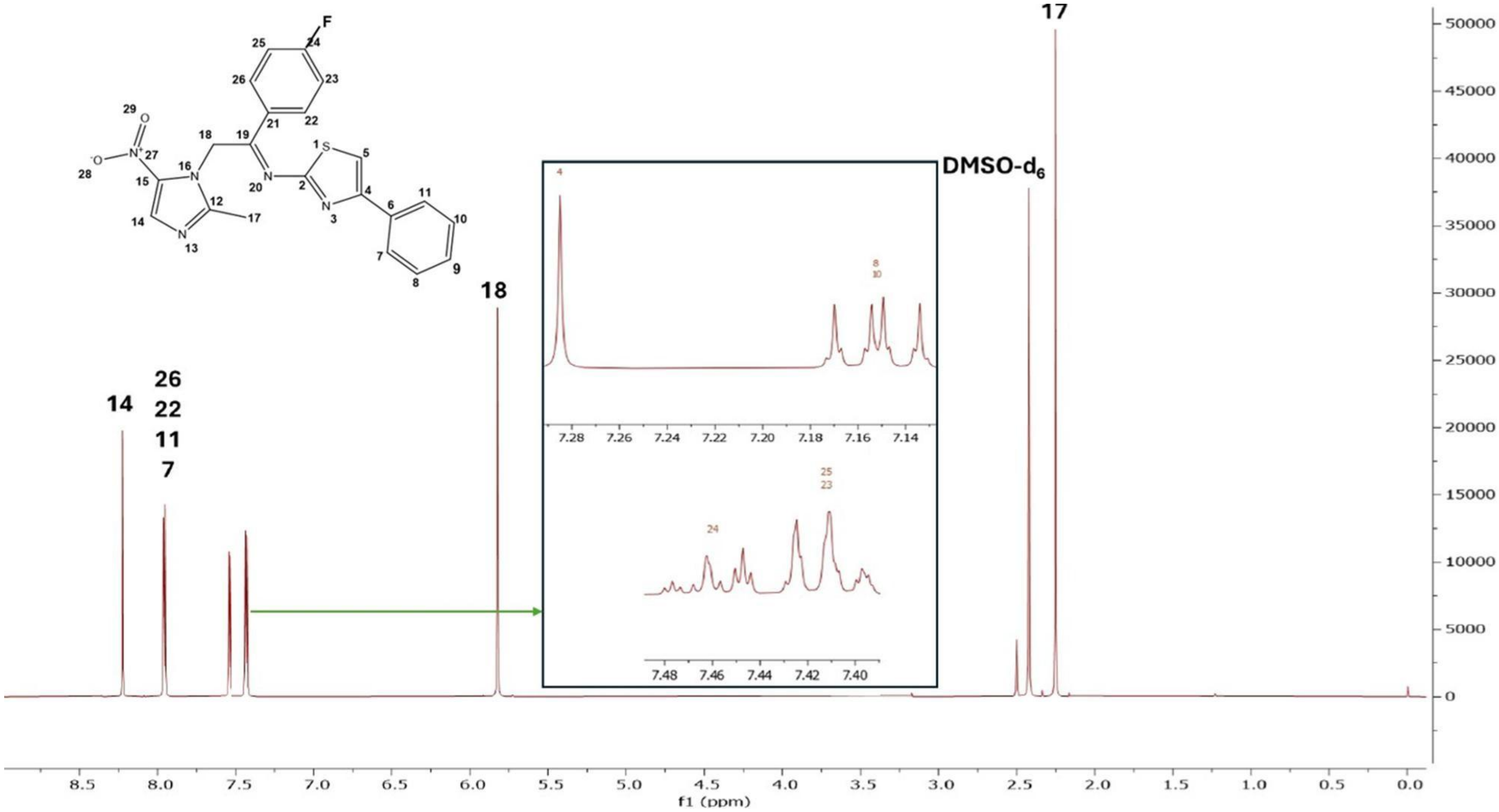

Figure S22. ^{1}H NMR spectrum of compound 3c (750 MHz, DMSO-d6).

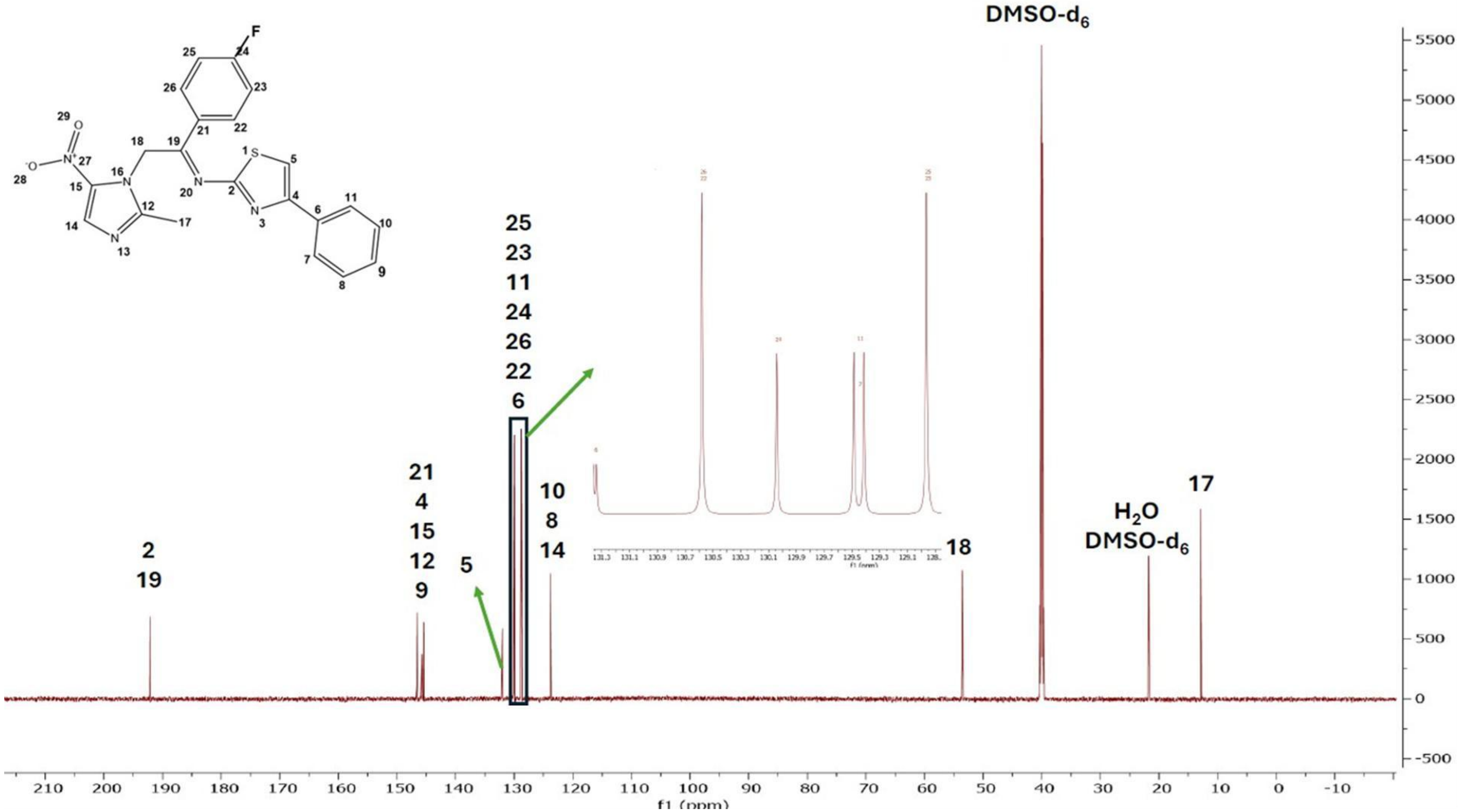

Figure S23. ^{13}C NMR spectrum of compound 3c (189 MHz, DMSO-d6).

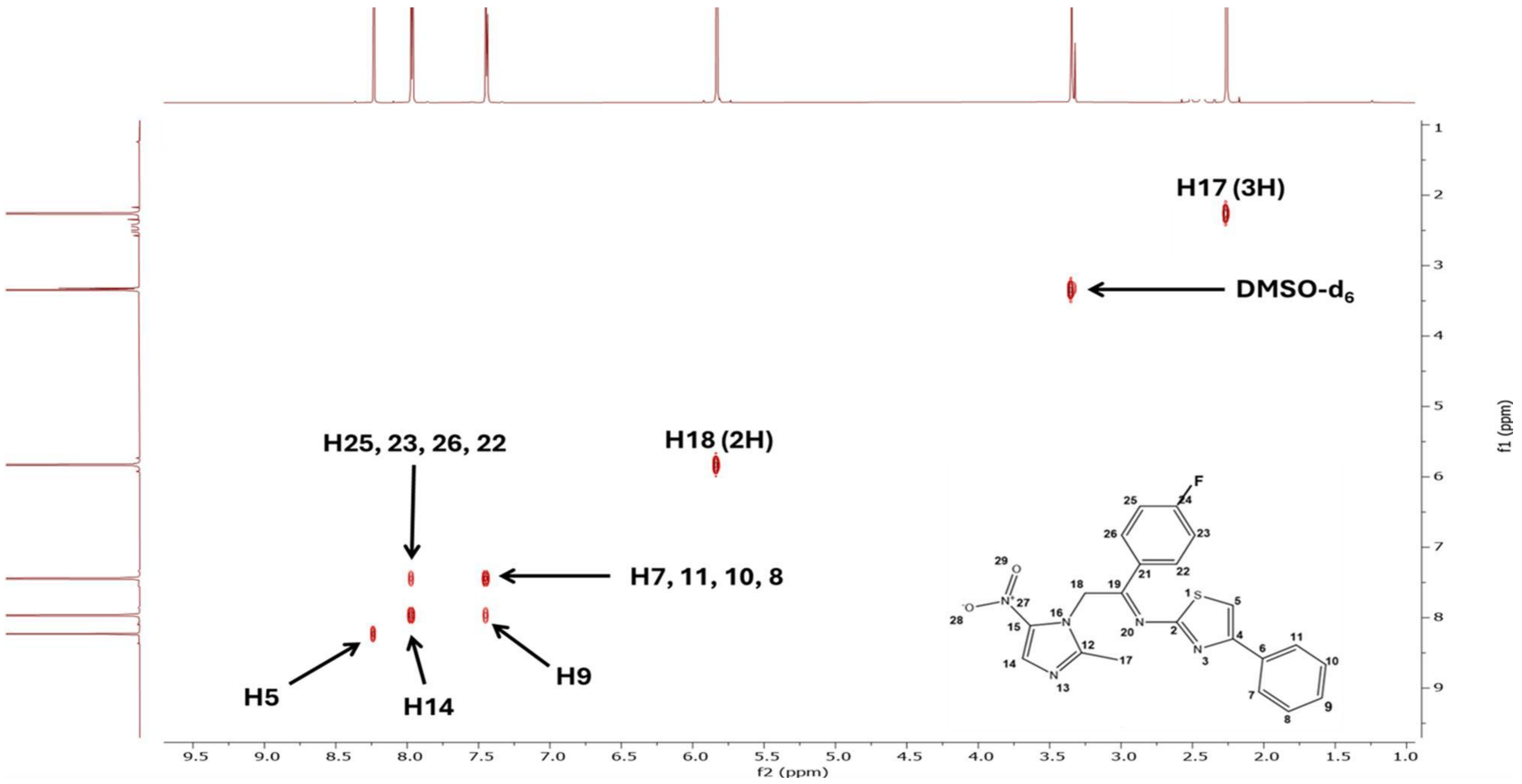

Figure S24. ^{1}H-^{1}H COSY spectrum of compound 3c (750 MHz, DMSO-d6).

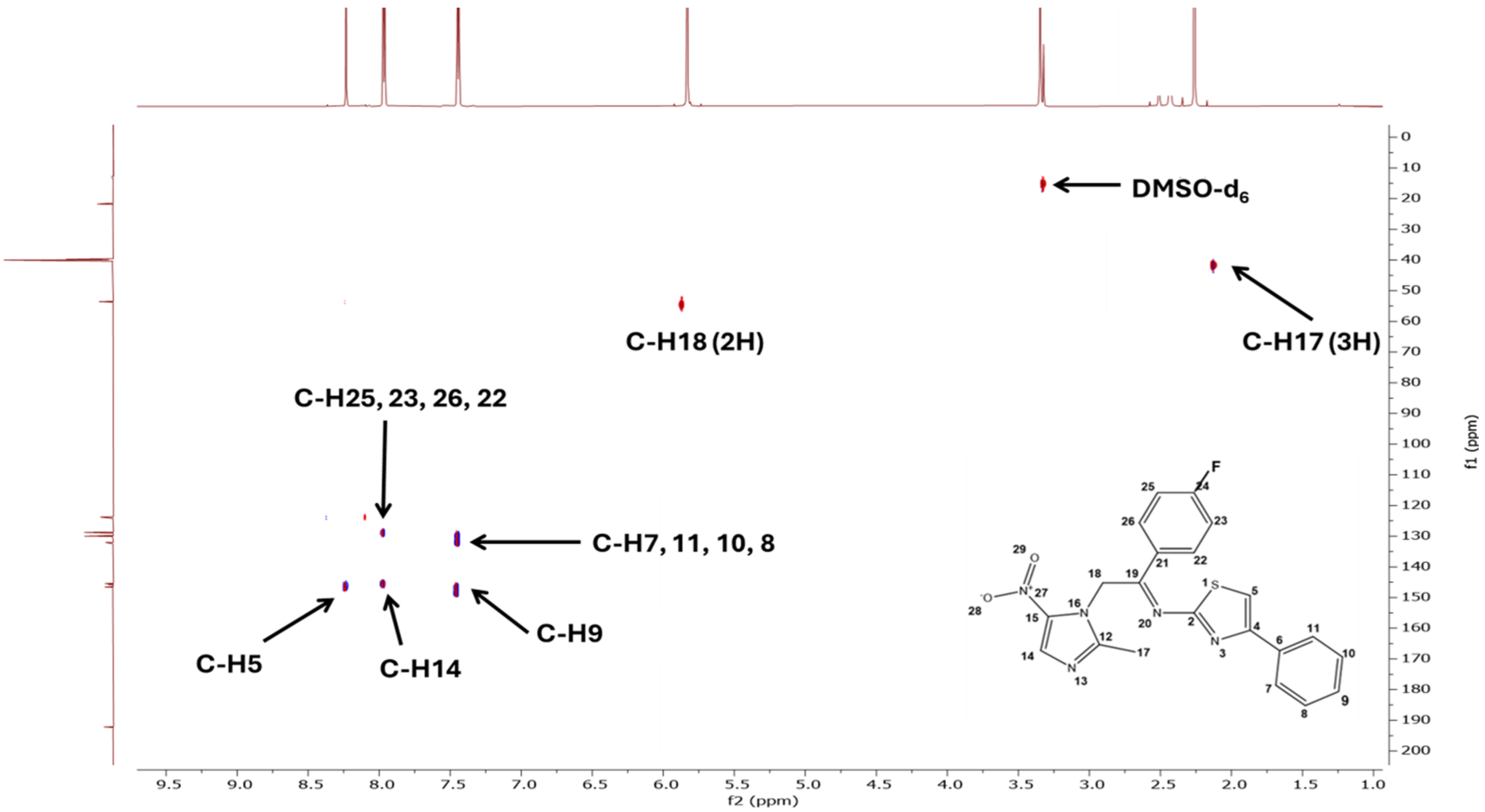

Figure S25. ^{1}H–^{13}C HMBC spectrum of compound 3c (750 MHz for ^{1}H, 189 MHz for ^{13}C, DMSO-d6).

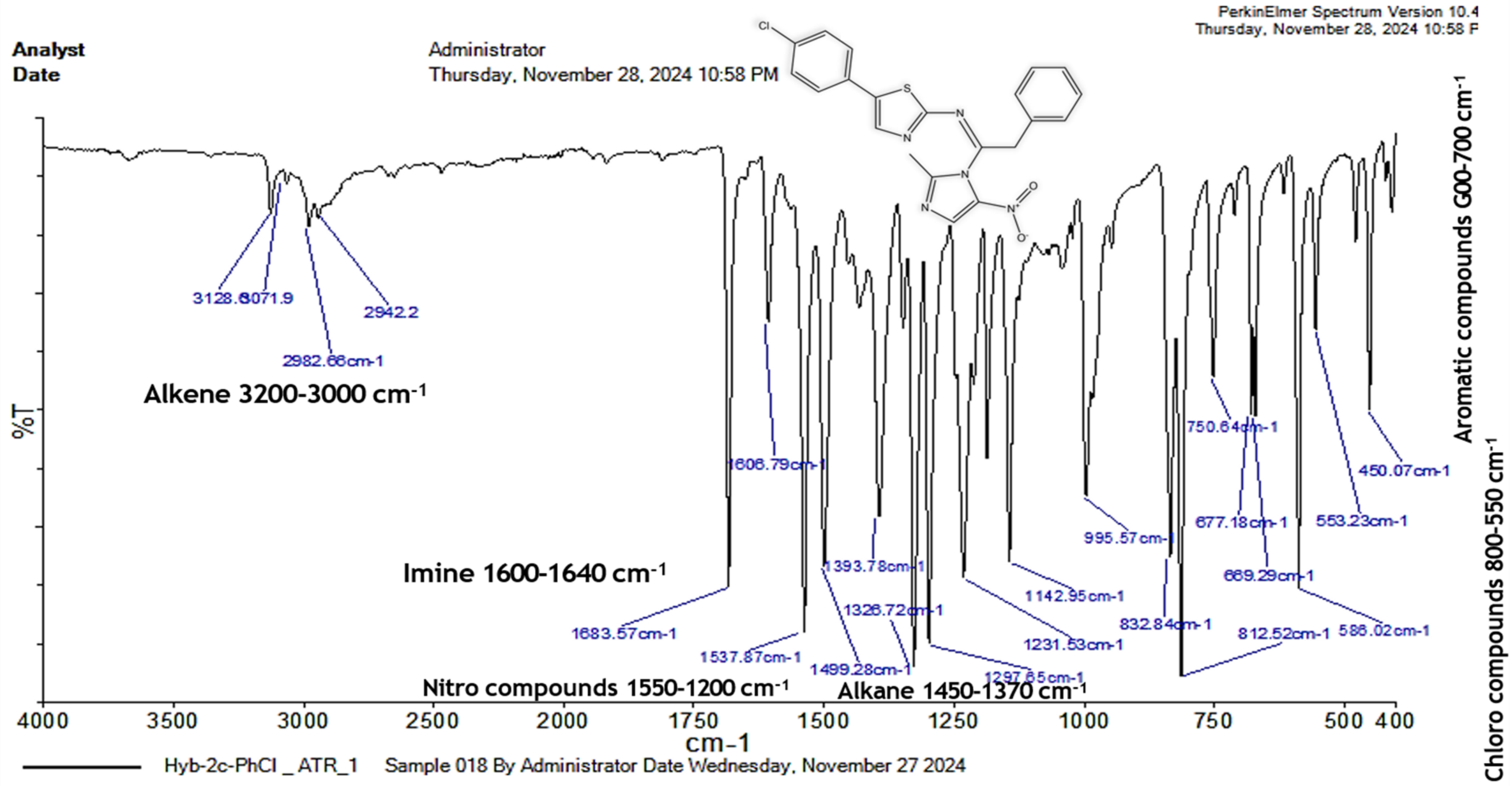

Figure S26. IR spectrum of compound 3d.

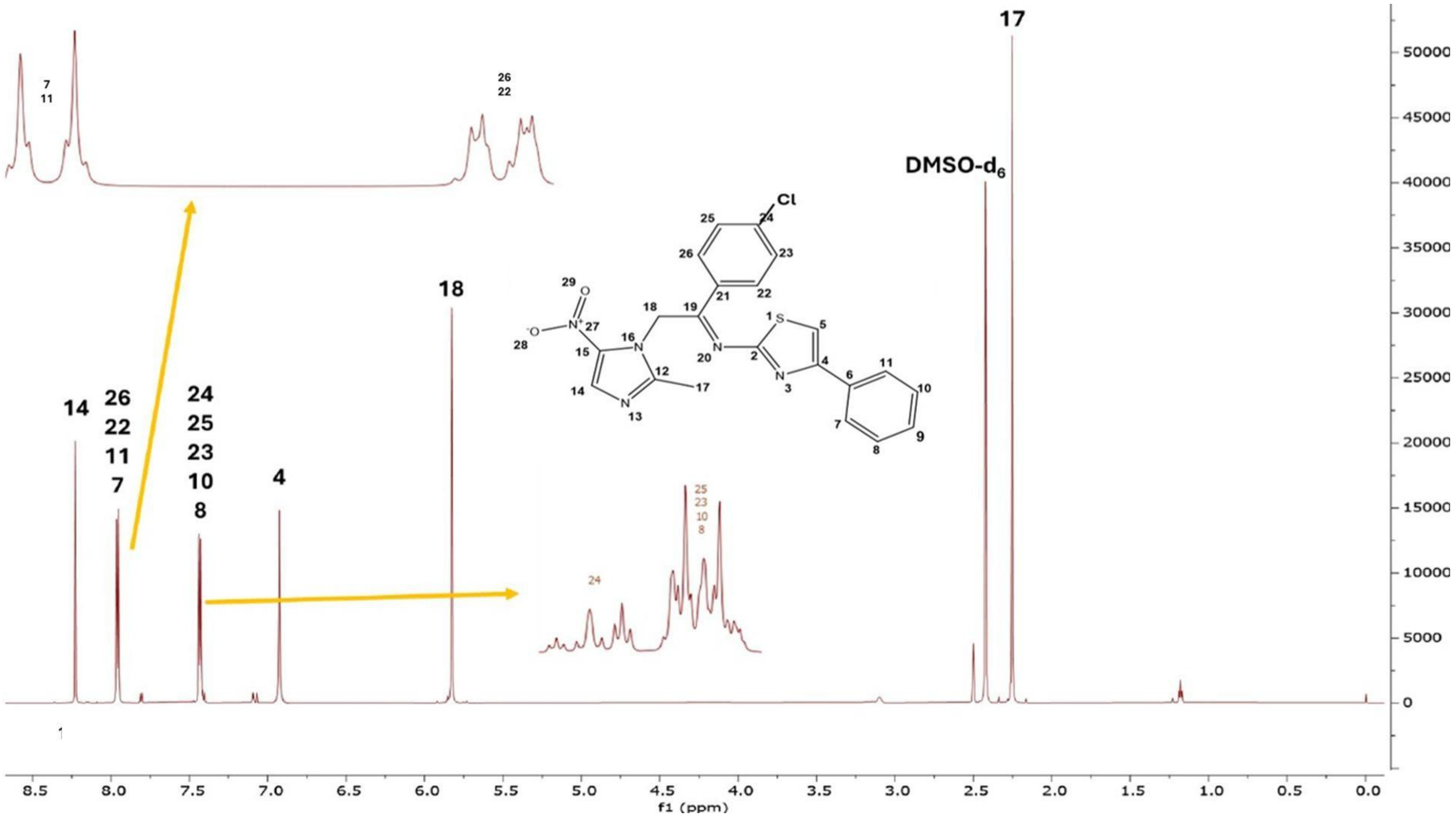

Figure S27. ^{1}H NMR spectrum of compound 3d (750 MHz, DMSO-d6).

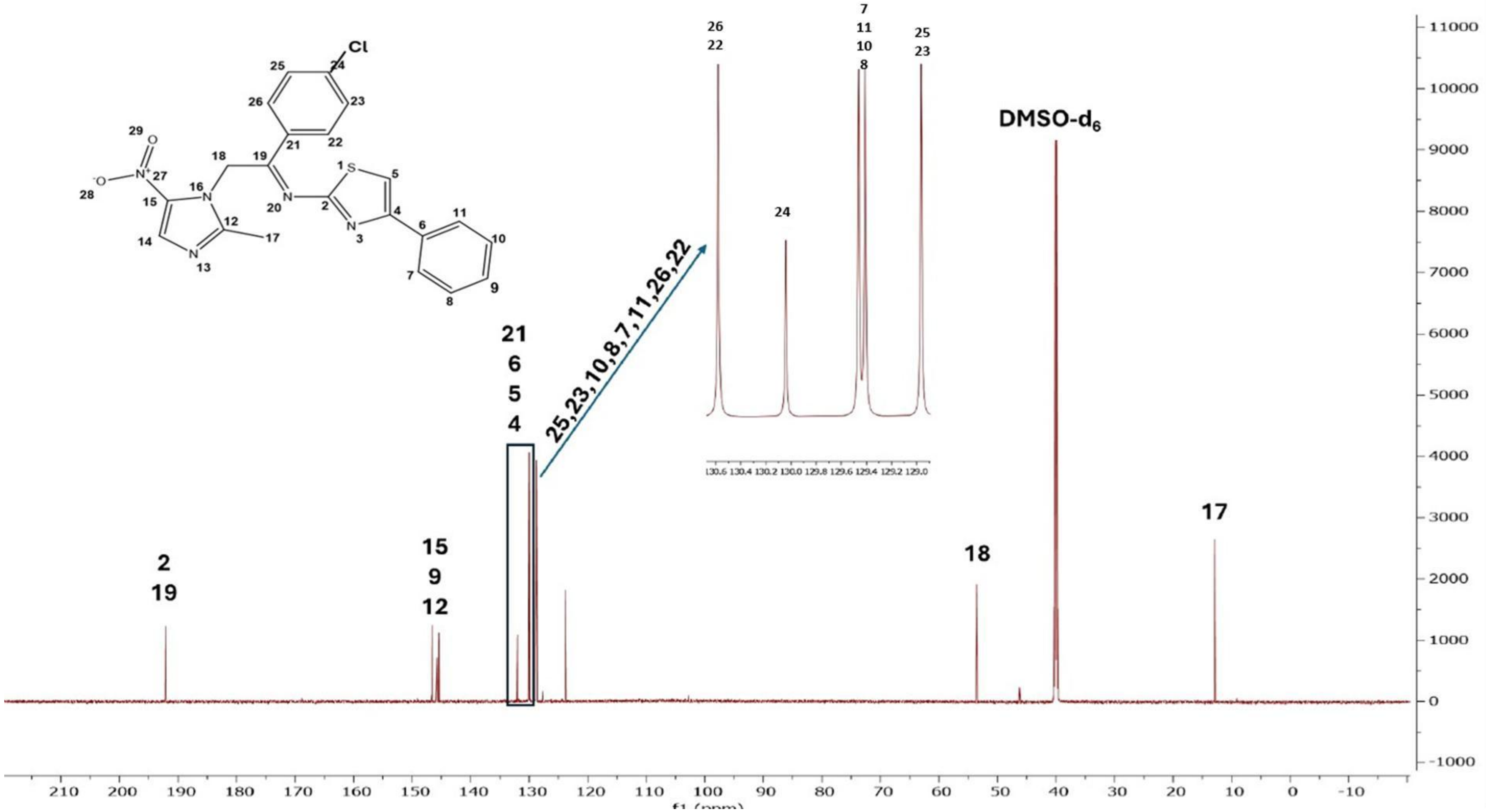

Figure S28. ^{13}C NMR spectrum of compound 3d (189 MHz, DMSO-d6)

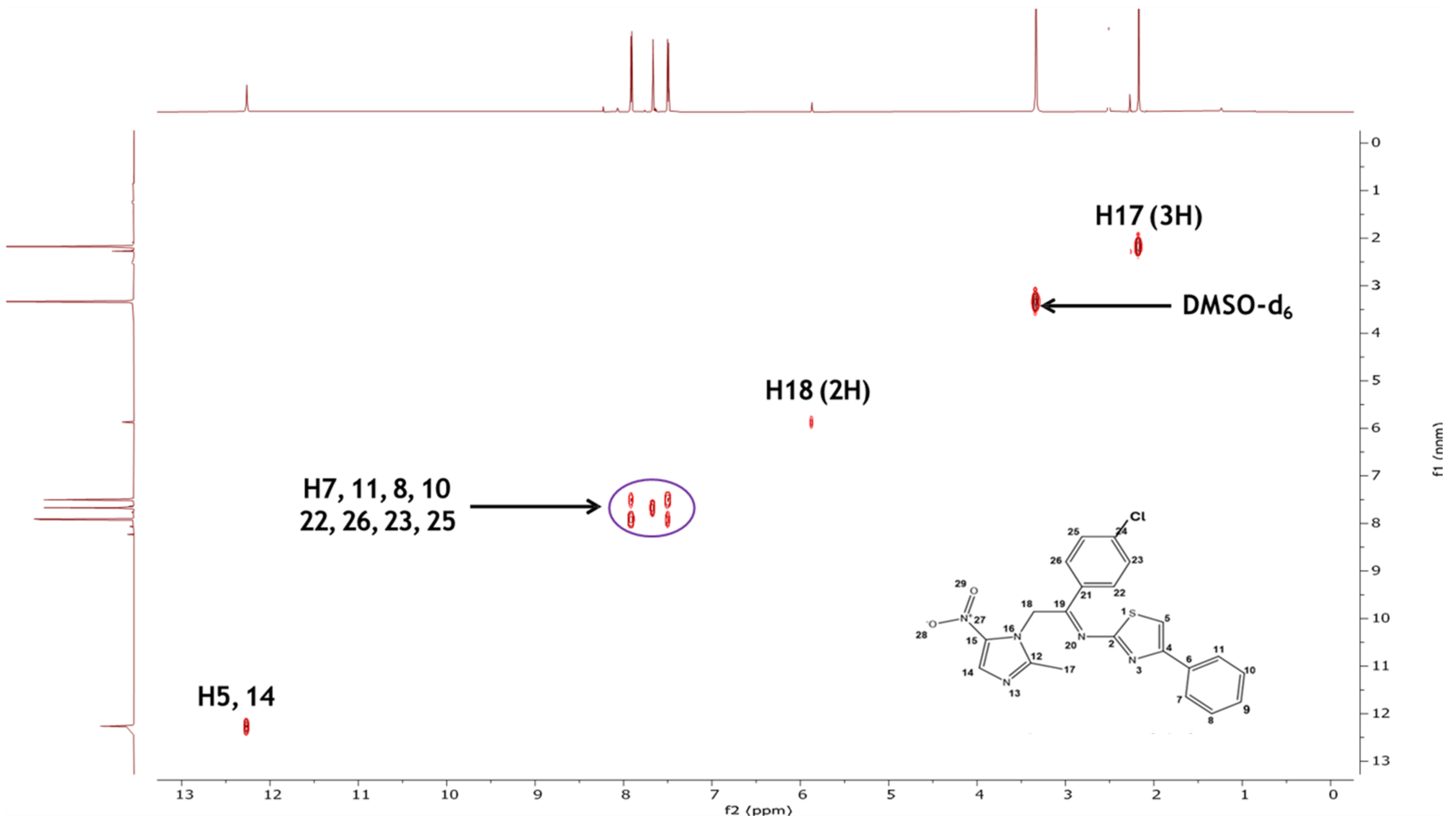

Figure S29. ^1H-^1H COSY spectrum of compound 3d (750 MHz, DMSO-d6)

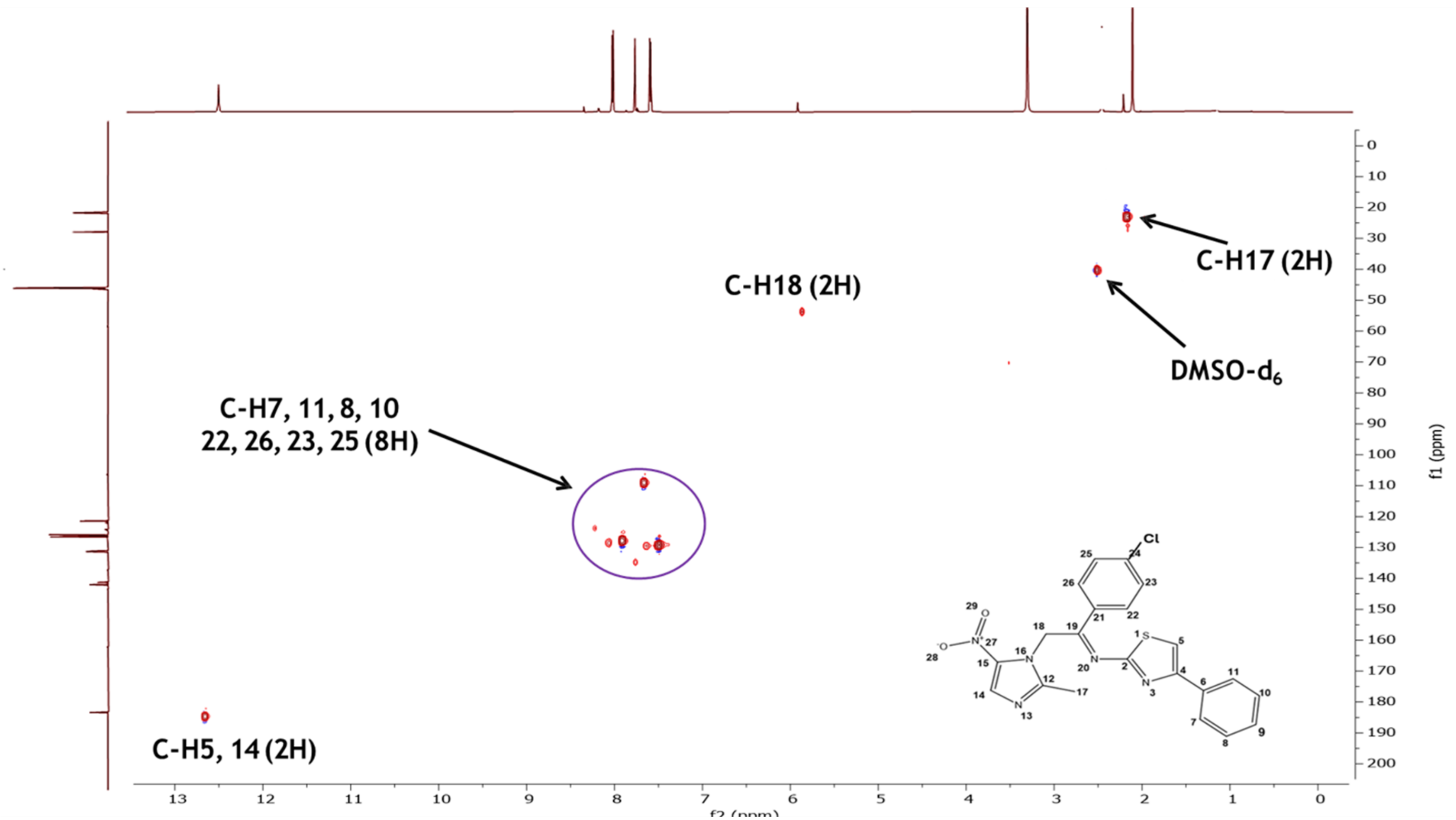

Figure S30. ^1H–^{13}C HMBC spectrum of compound 3d (750 MHz for ^1H, 189 MHz for ^{13}C, DMSO-d6).

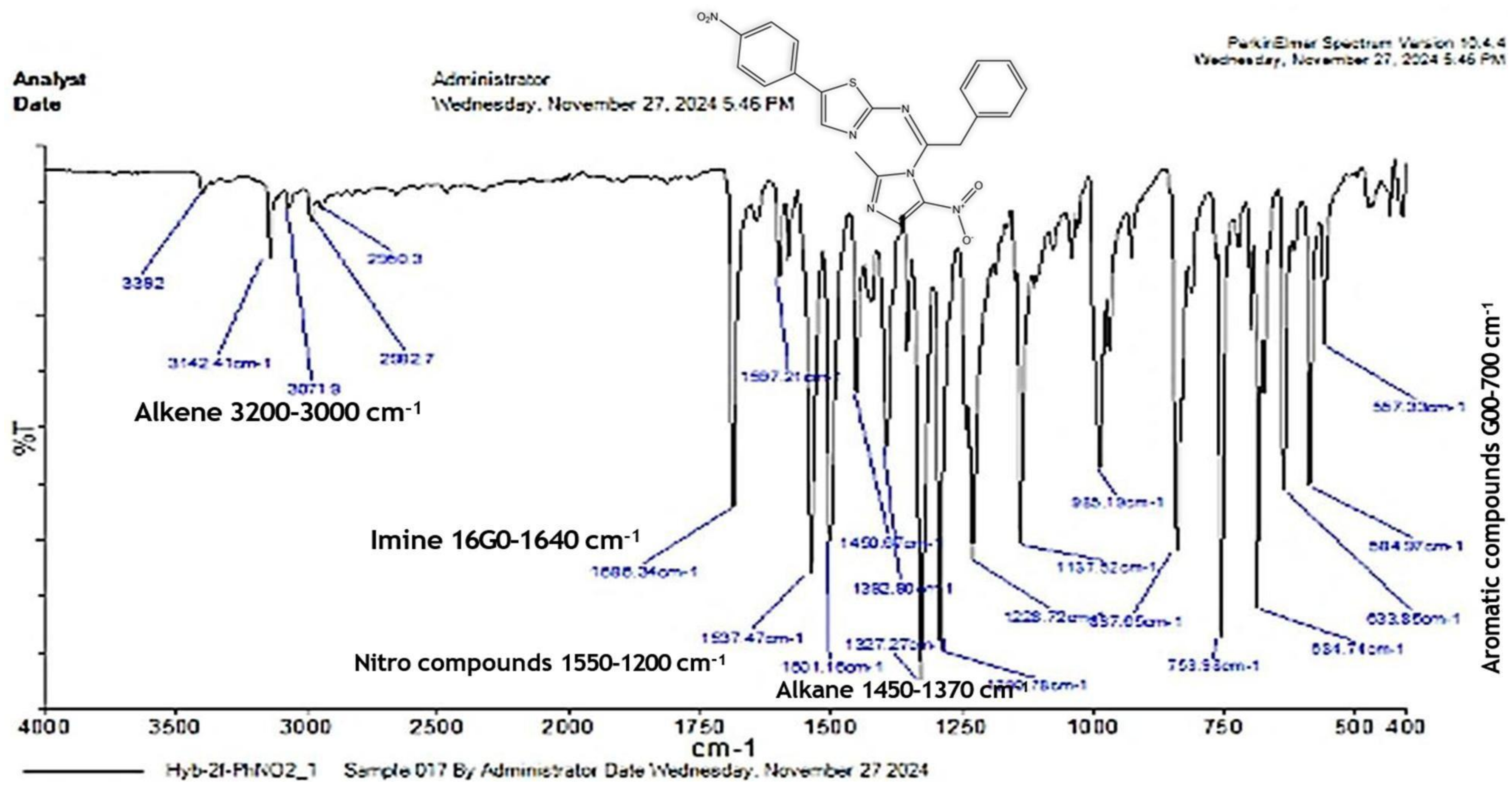

Figure S31. IR spectrum of compound 3f.

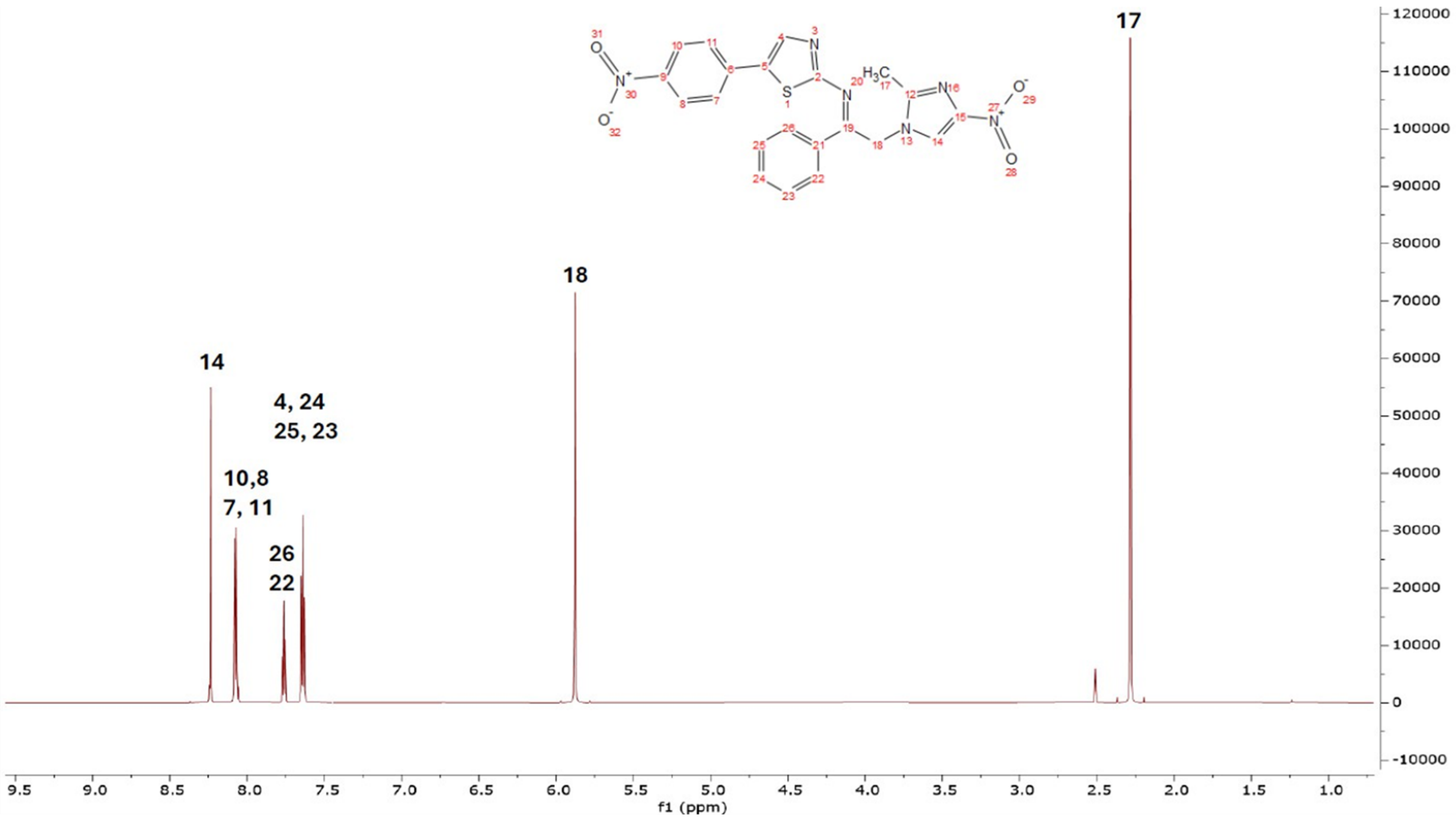

Figure S32. ^{1}H NMR spectrum of compound 3f (750 MHz, DMSO-d6)

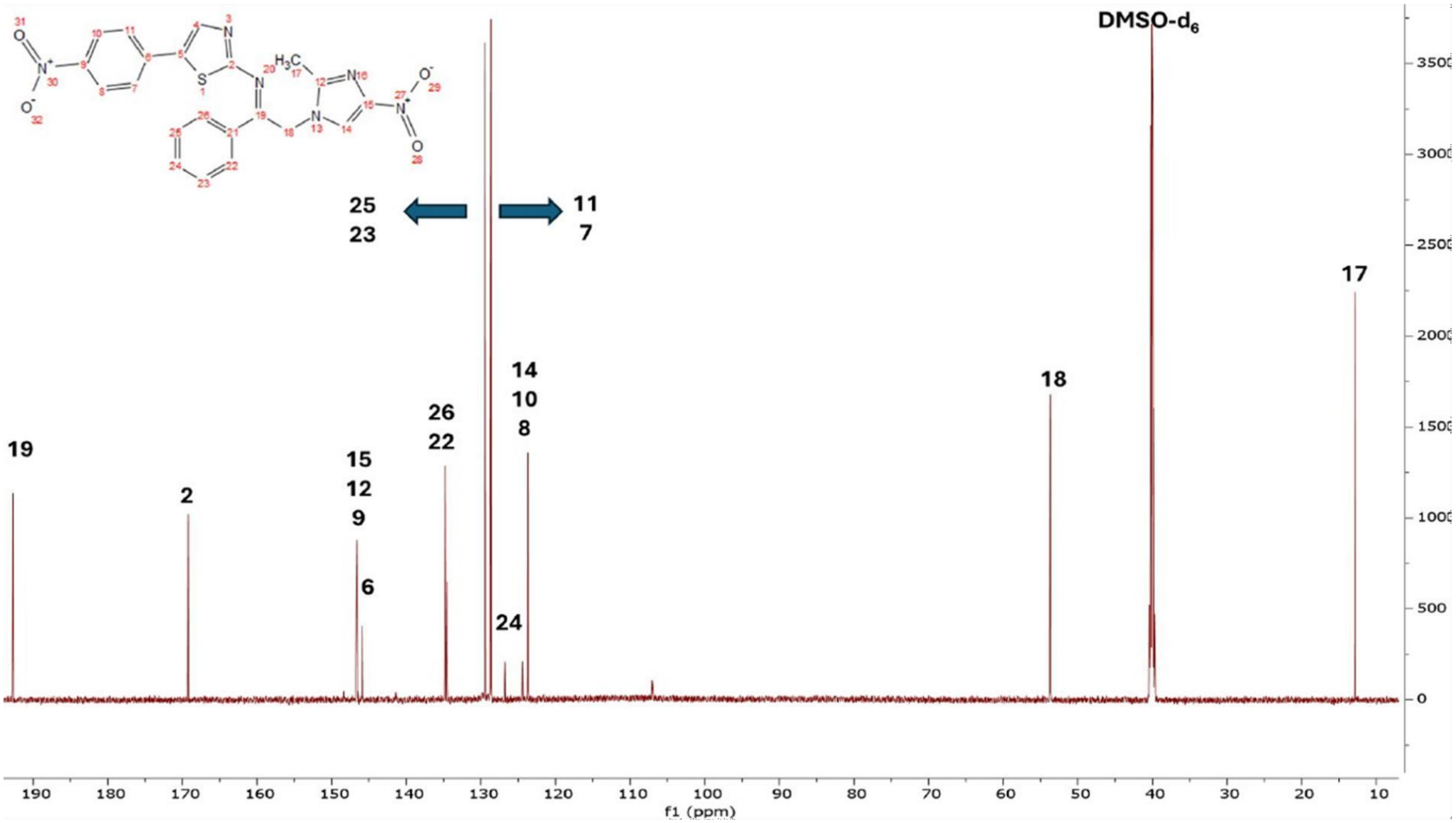

Figure S33. ^{13}C NMR spectrum of compound 3f (189 MHz, DMSO-d6)

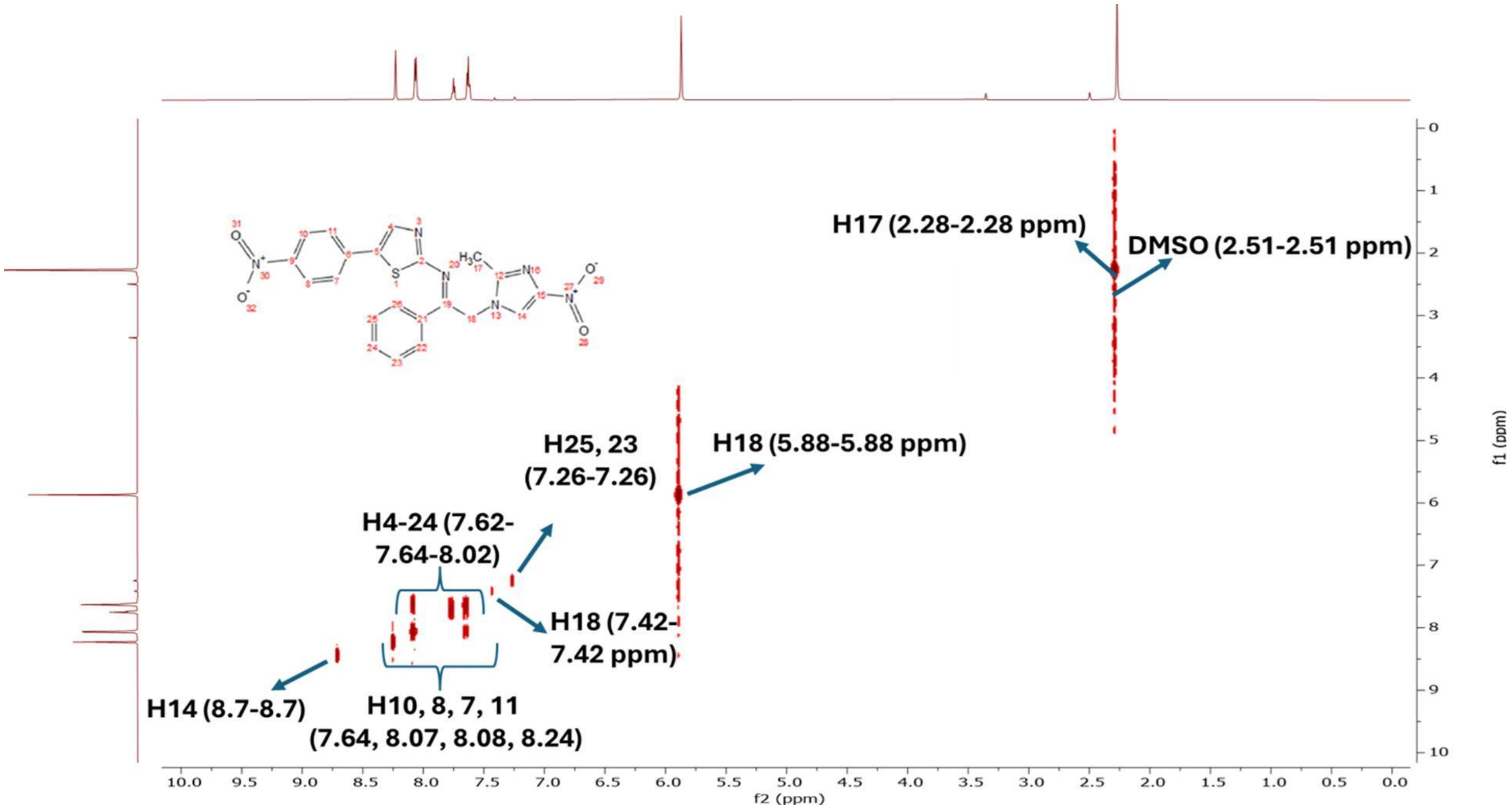

Figure S34. ^{1}H-^{1}H COSY spectrum of compound 3f (750 MHz, DMSO-d6)

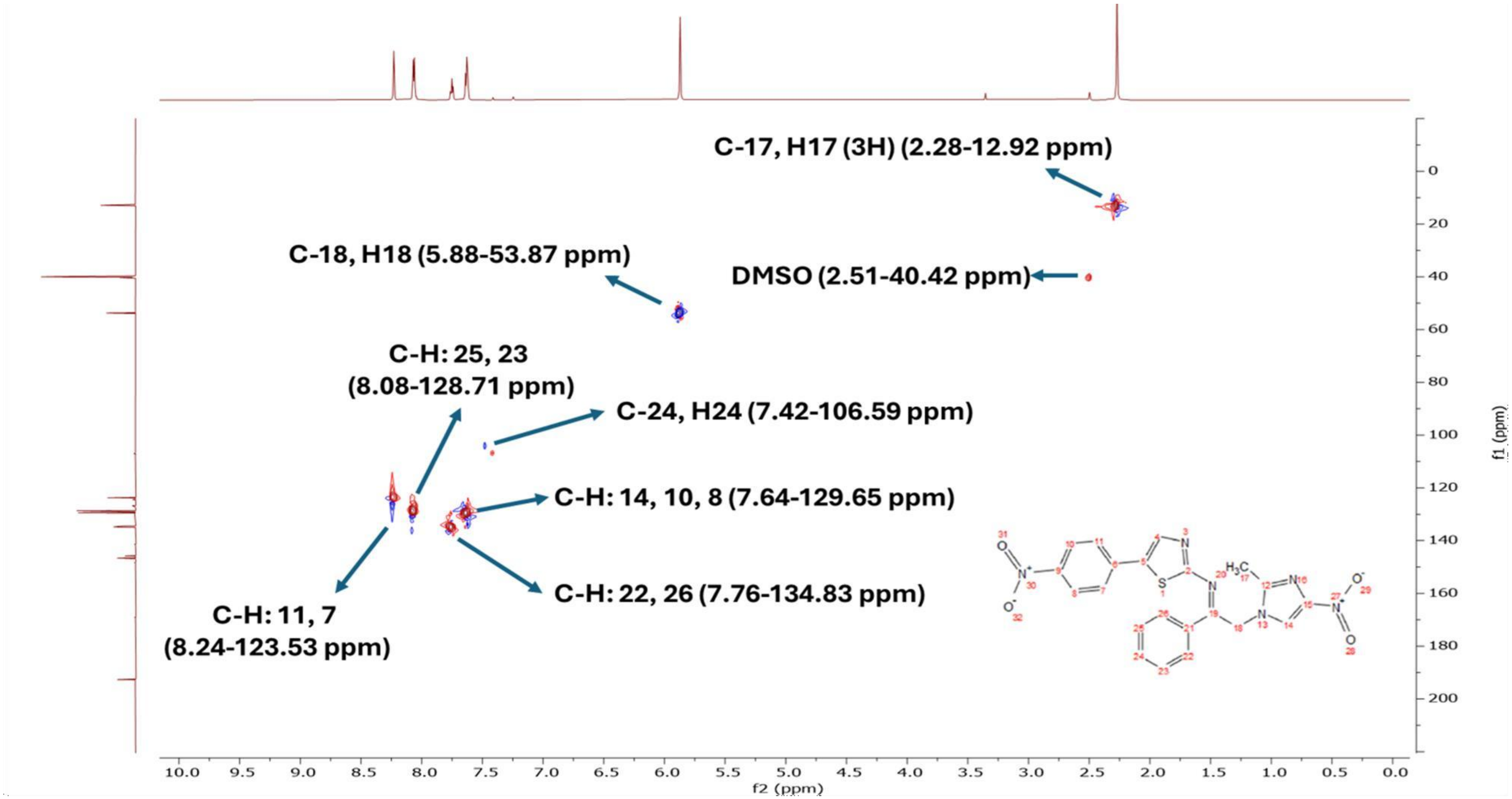

Figure S35. ^{1}H–^{13}C HMBC spectrum of compound 3f (750 MHz for ^{1}H, 189 MHz for ^{13}C, DMSO-d6).
